# A Hybrid Random Forest–SARSA Framework for Resting‐State EEG‐Based Parkinson's Disease Detection With Temporal Decision Refinement

**DOI:** 10.1002/brb3.71563

**Published:** 2026-07-09

**Authors:** Sanju S, S. Edward Rajan

**Affiliations:** ^1^ Department of Electrical and Electronics Engineering Rohini College of Engineering and Technology (Autonomous) Anjugramam Tamil Nadu India; ^2^ Department of Electrical and Electronics Engineering Mepco Schlenk Engineering College (Autonomous) Sivakasi Tamil Nadu India

**Keywords:** Parkinson's disease, Random Forest, resting‐state EEG, SARSA, subject‐wise validation, temporal decision refinement

## Abstract

Resting‐state electroencephalography (EEG) is an easily obtainable and noninvasive signal source for Parkinson's disease (PD) research. However, automatic PD classification from EEG is not a simple task, because EEG signals are noisy, non‐stationary, and vary considerably among individuals. This article presents a computationally lightweight two‐stage offline framework for window‐based PD and healthy‐control (HC) classification. In the first stage, a Random Forest classifier is used to generate the initial PD/HC prediction for each EEG window, along with the corresponding prediction confidence. In the second stage, SARSA is applied as a temporal decision‐refinement method. Instead of treating each EEG window as an isolated decision, SARSA uses the classifier confidence and the previously refined label to reduce sudden changes between neighboring window predictions. During training, the SARSA reward encourages correct PD/HC classification and applies a small penalty for unnecessary label switching. During testing, the learned SARSA policy is kept fixed and is used only to refine the prediction sequence of unseen subjects. The proposed framework was evaluated using the OpenNeuro ds002778 resting‐state EEG dataset. The Stage‐1 Random Forest model achieved 74.0%‐fold‐aggregated window‐level accuracy, while the final RF + SARSA framework improved the accuracy to 78.6%. For subject‐level evaluation, majority voting was applied to the refined window‐level predictions of each participant. The RF + SARSA framework achieved 77.4% subject‐level accuracy, with 24 correct predictions from 31 subjects and a 95% confidence interval of 60.2%–88.6%. McNemar's exact test showed no statistically significant subject‐level difference between Random Forest and RF + SARSA. Overall, the results suggest that SARSA‐based refinement can improve temporal consistency in offline EEG‐based PD/HC classification.

## Graphical abstract

The graphical abstract gives an overview of the proposed RF‐SARSA framework used for offline Parkinson's disease and healthy‐control classification from resting‐state EEG signals. The EEG recordings are first cleaned through basic preprocessing steps and then divided into 2‐second windows. From each window, 14 meaningful EEG features are extracted, covering band power, spectral ratio, statistical, Hjorth, and entropy‐based information. These features are given to the Random Forest model, which produces the first PD/HC prediction along with a confidence score. After this, SARSA refines the sequence of window‐level predictions by considering the classifier confidence and the previously refined label. This step helps reduce sudden changes between nearby EEG‐window decisions. Finally, majority voting combines the refined window predictions and produces the final subject‐level PD/HC result.

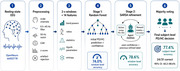

## 1 Introduction

Parkinson's disease is a slowly progressive neurological disorder that affects both motor and non‐motor functions. It is generally recognized through movement‐related symptoms such as tremor, muscle rigidity, bradykinesia, and postural instability. Still, these clinical signs alone do not completely explain the condition, because many patients also experience cognitive and behavioral changes that influence disease severity and vary from one individual to another (Bloem et al. 2021; Aarsland et al. 2021). Early detection is therefore not always straightforward, especially because the disease may appear differently in different patients during the initial stage. Due to these challenges, objective biomarker‐based assessment methods are gaining importance in Parkinson's disease evaluation. They provide measurable support for clinical judgment and may help in understanding the disease more clearly, especially when symptoms vary from one patient to another (Tolosa et al. 2021).

Electroencephalography (EEG) offers a noninvasive, repeatable, and comparatively affordable way to examine brain activity. In Parkinson's disease studies, resting‐state EEG has been used to observe changes in brain rhythms, spectral power, signal complexity, and connectivity patterns (Pappalettera et al. 2022; Chang et al. 2022; di Biase et al. 2023). Such EEG‐based measures can support the identification of differences between Parkinson's disease subjects and healthy controls, particularly when they are analyzed using appropriate machine‐learning methods (Aljalal et al. 2022; Kurbatskaya et al. 2023). Recent studies also suggest that resting‐state EEG features may reflect disease‐related neural alterations and can provide useful support for Parkinson's disease analysis (Anjum et al. 2024; Jibon et al. 2024). Earlier scalp EEG studies in Parkinson's disease have shown changes such as abnormal cortical synchrony, beta‐band activity alterations, and differences in waveform shape. These findings suggest that scalp EEG can provide useful information for studying disease‐related neural activity in Parkinson's disease (George et al. 2013; Swann et al. 2015; Jackson et al. 2019).

Machine‐learning and deep‐learning techniques are now commonly used for EEG‐based Parkinson's disease classification, mainly because they can identify useful patterns from complex EEG signals that may not be easily observed through manual inspection. Conventional models such as Support Vector Machine, k‐nearest neighbor, Decision Tree, and Random Forest are still useful because they are easier to implement, computationally lighter, and more interpretable for biomedical studies. However, many earlier works mainly focus on feature extraction or direct classification. Comparatively less attention has been given to the stability of predictions across consecutive EEG windows, although resting‐state EEG signals naturally change over time. This issue shows the need for a classification framework that does more than assign PD/HC labels. It should also maintain stable and consistent decisions across consecutive EEG windows. Recent research has also shown that deep‐learning models can identify useful EEG patterns for Parkinson's disease detection through CNN‐LSTM architectures, power spectral density features, and learned signal representations (Li et al. 2024; Obayya et al. 2023; Göker 2023). At the same time, feature‐based machine‐learning approaches and multiscale EEG models remain important because they can maintain useful diagnostic performance with lower model complexity (Wu et al. 2024; Qiu et al. 2024). Therefore, Random Forest is suitable for the present study because it can model nonlinear relationships among EEG features, reduce overfitting through ensemble learning, and provide more interpretable decision support than highly complex deep‐learning models.

A major difficulty in window‐level EEG classification is maintaining stable predictions over time. Resting‐state EEG signals are usually noisy, non‐stationary, and influenced by differences within and between subjects. Because of this, neighboring EEG windows from the same subject may sometimes produce inconsistent predictions (Singh and Bianchi 2024; Belhadi et al. 2025). Recent studies have shown that the length and position of EEG windows can influence the performance of machine‐learning models (Wosiak et al. 2025). This is an important point in resting‐state EEG analysis, as the signal does not remain uniform throughout the recording. Even nearby EEG windows may show small variations, and these changes can sometimes lead to unstable predicted labels. To reduce such sudden label changes, methods such as majority voting, hysteresis thresholding, and HMM/Viterbi decoding are commonly used. These methods can smooth the prediction sequence to some extent, but they mostly depend on neighboring window outputs or fixed transition rules (Chen et al. 2024; Heintz et al. 2025). Since EEG signals naturally vary over time, rule‐based methods may not always manage repeated prediction fluctuations effectively. This limitation supports the need for a learning‐based temporal refinement method that can use both classifier confidence and previous decision information to produce more consistent EEG‐window predictions. A learning‐based temporal refinement method is more suitable in this context because it can use classifier confidence and previous decision information to improve the consistency of predictions across successive EEG windows.

Based on the above motivation, this study presents a lightweight hybrid Random Forest–SARSA framework for Parkinson's disease detection using resting‐state EEG signals. Recent EEG‐based studies show that machine‐learning methods can support automatic Parkinson's disease detection, while recent reviews also emphasize the need for transparent models and reproducible validation in Parkinson's disease research (Latifoğlu et al. 2024; Shokrpour et al. 2025). In the proposed framework, Random Forest is used as the first‐stage classifier to generate window‐level Parkinson's disease and healthy‐control predictions from interpretable EEG features. SARSA is then applied as a second‐stage offline temporal refinement method to improve the stability of the prediction sequence. Although reinforcement‐learning methods have recently been studied in EEG signal modeling and adaptive DBS‐related research, SARSA is not used as a DBS stimulation controller in this study (Zhang et al. 2024; Zhao et al. 2024; Ravivarapu et al. 2025). Its action space is limited to refined Parkinson's disease and healthy‐control decision labels, and its role is restricted to improving temporal consistency in EEG‐based classification.

The main gap addressed in this study is the need for a reproducible framework that combines interpretable EEG feature‐based classification with learned temporal decision refinement under subject‐wise validation. Recent EEG‐based Parkinson's disease studies have introduced both feature‐based and deep‐learning methods, but most of them mainly aim to improve classification accuracy and do not directly address unstable predictions across EEG windows (Lal et al. 2024; Fernandez et al. 2025; Wang et al. 2025). Advanced deep‐learning models have also shown promise for Parkinson's disease detection, but they may increase model complexity and need careful validation before any clinical interpretation is made (Bunterngchit et al. 2025; Oh et al. 2025). To address this research gap, the present study brings together an interpretable Random Forest classifier and a SARSA‐based temporal refinement strategy. The evaluation is carried out using a subject‐wise validation approach. In this setup, all EEG windows from one participant are kept either in the training set or in the testing set, but not in both. This separation is important because it reduces the risk of subject‐level data leakage and gives a more realistic idea of how the model performs on unseen participants.

The scope of this work is also kept clear. The study focuses only on offline resting‐state EEG‐based classification of Parkinson's disease and healthy‐control subjects. SARSA is used only to refine the sequence of PD/HC decisions produced by the Random Forest model. It does not estimate cognitive status, simulate closed‐loop DBS activity, or suggest any stimulation‐related decision. In simple terms, SARSA works as a temporal decision‐refinement step. It takes the Random Forest output and helps make window‐level PD/HC predictions more consistent across consecutive EEG segments.

The main contributions of this study are listed below:

1. A reproducible preprocessing workflow is developed for resting‐state EEG‐based Parkinson's disease detection using the BIDS‐formatted OpenNeuro ds002778 dataset.
2. A compact 14‐dimensional EEG feature set is extracted from spectral, statistical, and complexity‐related signal characteristics to support interpretable classification.
3. Random Forest is used as the first‐stage classifier to produce window‐level Parkinson's disease and healthy‐control predictions under subject‐wise validation.
4. SARSA is applied as a temporal refinement stage to improve the stability of window‐level predictions by using classifier confidence and previous decision information.
5. Standard temporal smoothing methods, including majority voting, hysteresis thresholding, and HMM/Viterbi decoding, are compared with the proposed SARSA‐based refinement approach.
6. The interpretation of the framework is restricted to offline EEG‐based Parkinson's disease detection with temporal decision refinement, since the dataset does not include cognitive scores, fluctuation labels, DBS stimulation settings, or stimulation‐response measurements.

The rest of the article is organized as follows. Section 2 explains the proposed methodology, including EEG preprocessing, feature extraction, Random Forest classification, and SARSA‐based temporal decision refinement. Section 3 presents the experimental setup, evaluation protocol, results, temporal baseline comparison, and discussion. Section 4 concludes the article by summarizing the main findings, limitations, and future research directions.

## 2 Proposed Methodology

This study introduces a two‐stage hybrid framework for detecting Parkinson's disease from resting‐state EEG signals with improved decision stability over time. The proposed workflow includes EEG preprocessing, interpretable feature extraction, Random Forest–based classification, and SARSA‐based temporal refinement. In the first stage, the extracted EEG features from each window are used by the Random Forest classifier to identify whether the signal pattern belongs to the Parkinson's disease group or the healthy‐control group. In the second stage, SARSA is applied only as a temporal refinement technique. It considers the classifier confidence and the previous decision pattern to reduce sudden changes in window‐level predictions. Therefore, the reinforcement‐learning component of this study is not presented as a DBS stimulation controller. It is used only to improve prediction consistency in an offline EEG classification framework and should not be interpreted as a clinically validated DBS control method.

The proposed framework is kept within a clear offline EEG classification setting. It is mainly intended to distinguish Parkinson's disease subjects from healthy‐control subjects using resting‐state EEG signals, and then to make the window‐level decisions more stable over time. The method does not try to identify cognitive states, simulate DBS stimulation, or decide any stimulation‐related parameter. In the first stage, the Random Forest model gives an initial PD/HC prediction along with a confidence score for each EEG window. After that, SARSA works as a refinement step. It uses the confidence value and the previously refined decision to reduce sudden label changes between nearby EEG windows. So, in this study, SARSA should be understood only as a temporal decision‐refinement method, not as a clinical control policy or DBS‐related decision system.

In the first stage, the extracted EEG features are given to the Random Forest classifier, which produces an initial PD/HC prediction for every EEG window. This step keeps the classification process simple, transparent, and easier to explain, which is useful in biomedical research where the model output should be understandable. The second stage has a different purpose. SARSA is not used as a separate classifier; instead, it works on the sequence of Random Forest predictions and refines them over time. It considers the classifier confidence score and the previously refined decision to reduce sudden label changes between neighboring EEG windows.

Overall, the proposed methodology offers a clear, reproducible, and practical approach for detecting Parkinson's disease from resting‐state EEG signals. The framework brings together EEG preprocessing, interpretable feature extraction, Random Forest classification, and SARSA‐based temporal refinement within one organized pipeline. This design helps improve prediction stability while keeping the model lightweight and easy to interpret.

The motivation for this two‐stage design comes from the natural variability of resting‐state EEG signals. EEG recordings are noninvasive and practical to collect, but they are also noisy and different across subjects. Therefore, Random Forest is first used to obtain stable window‐level PD/HC predictions, and SARSA is then used to improve the consistency of these predictions over time. Since the work is evaluated using a public resting‐state EEG dataset, the framework remains reproducible and suitable for offline experimental validation.

**FIGURE 1:**
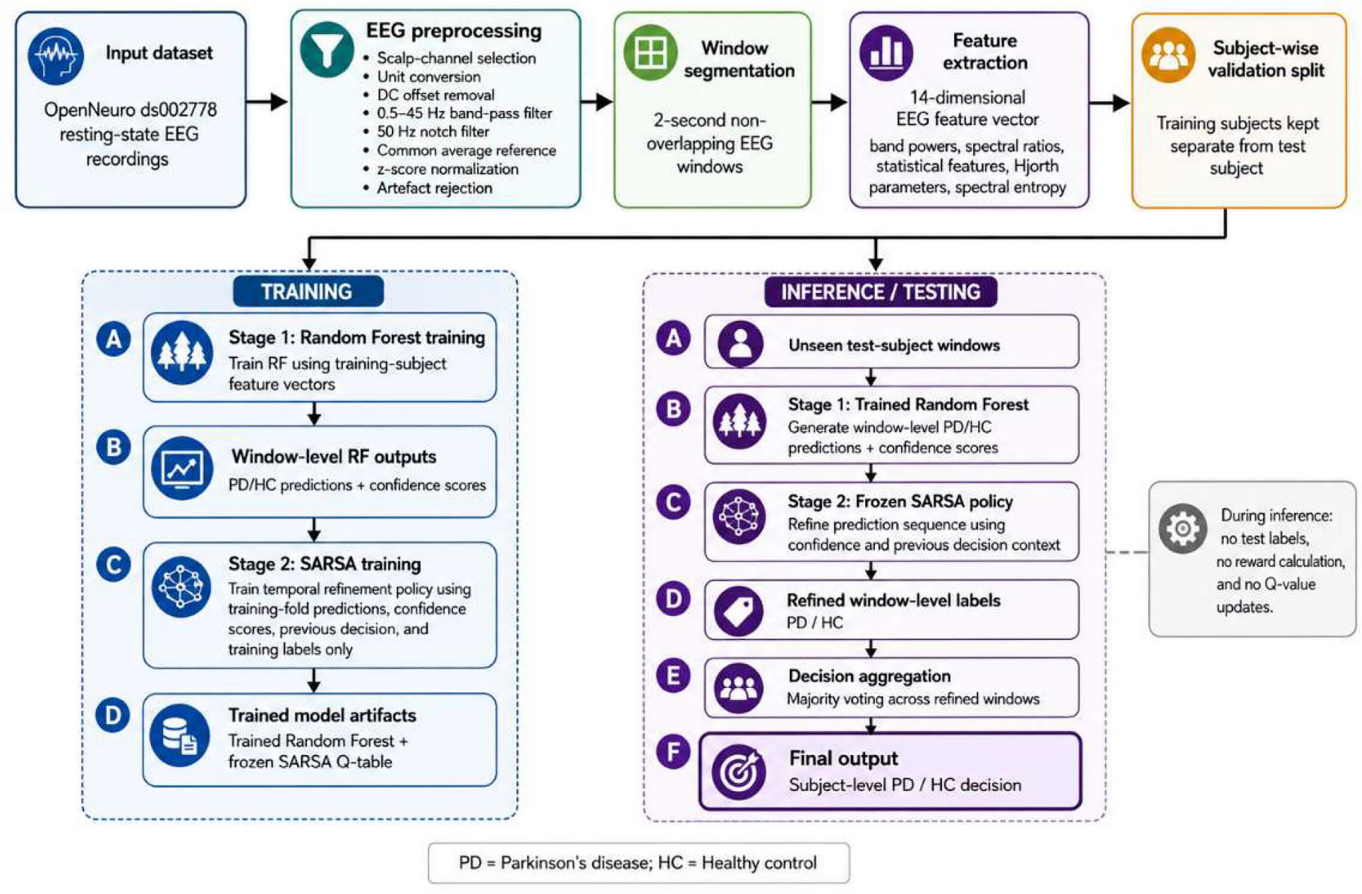
Overall workflow of the proposed Random Forest–SARSA framework for resting‐state EEG‐based Parkinson's disease detection with temporal decision refinement.

Figure 1 presents the overall workflow of the proposed RF + SARSA framework. The OpenNeuro ds002778 resting‐state EEG recordings are first preprocessed, divided into 2‐second windows, and transformed into 14‐dimensional feature vectors. A subject‐wise validation strategy is then applied so that EEG windows from the same subject are not shared between training and testing. During training, the Random Forest model learns window‐level PD/HC classification, while SARSA learns a temporal refinement policy using only training‐fold predictions, confidence scores, previous decisions, and labels. During testing, the trained Random Forest model and the fixed SARSA policy are applied to unseen subjects. At this stage, test labels are not used, rewards are not calculated, and Q‐values are not updated. Finally, the refined window‐level decisions are combined through majority voting to obtain the subject‐level PD/HC result.

### 2.1 Input EEG Dataset and Signal Representation

The proposed framework uses multichannel EEG recordings obtained from a publicly available Parkinson's disease dataset. EEG was chosen because it is noninvasive, easy to record, and suitable for analyzing resting‐state brain activity. In this study, EEG signals are used only for PD/HC classification and temporal decision refinement. This makes the method reproducible and suitable for subject‐wise evaluation, where the model is tested on unseen participants.

Let the EEG recording of a subject be represented as a multichannel signal:

(1) $$X\left(n\right)=\left\{x_{1}\left(n\right),x_{2}\left(n\right),\text{…},x_{C}\left(n\right)\right\},n=1,2,\text{…},N,$$

where C is the number of EEG channels, N is the total number of time samples, and $x_{c}\left(n\right)$ denotes the EEG amplitude of the *c*th channel at sample n. Since raw EEG recordings may include baseline drift, power‐line noise, physiological artifacts, and other unwanted variations, preprocessing is required before feature extraction. A preprocessing function P(·) is applied to obtain the cleaned EEG signal:

(2) $$\tilde{X}\left(n\right)=P\left(X\left(n\right)\right),$$

where P(·) includes band‐pass filtering, notch filtering, re‐referencing, normalization, and artifact handling. After preprocessing, the cleaned EEG signal is divided into fixed‐length windows of duration $T_{w}$:

(3) $$\;X_{t}=\tilde{X}\;\left(\tau_{t}:\tau_{t}+T_{w}-1\right),t=1,2,\text{…},K,$$

where $X_{t}$ represents the preprocessed EEG window at time index t, $\tau_{t}$ is the starting sample of the *t*th window, $T_{w}$ is the window length, and K is the total number of EEG windows. In this work, each $X_{t}$ corresponds to one 2‐second nonoverlapping EEG segment.

This window‐based representation helps the model analyze short‐term changes in resting‐state EEG signals. It also keeps the temporal order of EEG windows, which is needed for SARSA‐based decision refinement. Therefore, the EEG input, preprocessing, and fixed‐window segmentation together provide a clear and consistent basis for feature extraction, PD/HC classification, and temporal refinement.

Window‐based segmentation plays a useful role in the proposed framework, as it changes a continuous EEG recording into smaller and more manageable decision intervals. This makes the analysis more practical, because resting‐state EEG signals do not remain fully stable throughout the recording. By looking at short EEG segments, the model can capture local signal variations that may be missed when the whole recording is considered as one long signal. In the present study, each EEG window is treated as one PD/HC classification interval. The ordered sequence of these windows is also helpful for the SARSA stage, since SARSA needs previous decision information to refine the next prediction. In this way, window‐based segmentation supports smoother temporal decision refinement and improves the reliability of the model under subject‐wise evaluation.

This section adopts multichannel EEG as the input signal because it offers a noninvasive and scalable neural feedback source for modeling EEG‐based temporal decision refinement without relying on invasive recordings or specialized sensing hardware. Representing EEG as a structured multichannel time series and applying a standard preprocessing operator reduces common artifacts (drift, power‐line noise and physiological interference), which supports cleaner and more comparable signals across subjects. The window‐based representation is included to convert continuous EEG into discrete decision intervals, which is essential for two reasons: it enables time‐localized state estimation in a clinically realistic sensing cycle, and it provides the sequential state transitions required for reinforcement learning in Stage 2. Overall, this representation improves reproducibility, supports subject‐wise evaluation, and forms a practical foundation for feature extraction and adaptive decision modeling in the proposed framework.

### 2.2 EEG Preprocessing and Window‐Based Segmentation

EEG is used as the primary input signal in the proposed framework because it provides a noninvasive and temporally rich representation of brain activity and is widely employed in both clinical and research settings. However, raw EEG recordings are not directly suitable for learning‐based modeling due to the presence of artifacts, noise, and non‐stationary dynamics. Common noise sources in EEG include baseline drift, power‐line interference, physiological artifacts, and recording‐related variations. These unwanted components can disturb the EEG patterns needed for PD/HC classification. Therefore, systematic preprocessing is applied before feature extraction to obtain a cleaner and more reliable signal representation.

Let the raw multichannel EEG signal be represented as X(t). A preprocessing operator P(·) is applied to obtain the cleaned EEG signal:

(4) $$\tilde{X}\left(t\right)=P\left(X\left(t\right)\right),$$

where P(·) includes band‐pass filtering, notch filtering, re‐referencing, normalization, and artifact handling. These preprocessing operations are applied to minimize unwanted signal variations while preserving the physiologically meaningful EEG components needed for later analysis. After this stage, the cleaned EEG signal is divided into short fixed‐length windows of duration $T_{w}$:

(5) $$X_{t}=\tilde{X}\;\left(\tau_{t}:\tau_{t}+T_{w}-1\right),t=1,2,\text{…},K$$

where $X_{t}$ denotes the preprocessed EEG window at time index t, $\tau_{t}$ is the starting sample of the *t*th window, $T_{w}$ is the window length, and K is the total number of windows. In this study, each window represents a 2‐second EEG segment without overlap.

Window‐based segmentation is useful because it converts continuous EEG recordings into smaller analysis units that are easier to handle. This allows short‐term variations in resting‐state EEG activity to be examined more clearly. At the same time, the temporal order of the windows is maintained, which is necessary for SARSA‐based refinement. Therefore, preprocessing and fixed‐window segmentation provide a steady base for the next stages of the study, including feature extraction, PD/HC classification, and temporal decision refinement.

### 2.3 Feature Extraction: Interpretable EEG Features

After preprocessing and segmentation, each EEG window is represented using a small but meaningful set of numerical features. This step is important because raw EEG signals, in their original form, are usually large, noisy, and difficult to use directly for classification. Instead of passing the complete signal to the model, the useful signal characteristics are extracted and converted into a compact feature representation. This makes the input easier to handle and also keeps the classification process more interpretable. In the present study, these extracted features describe the main patterns present in each EEG window before applying the Random Forest classifier and SARSA‐based temporal refinement.

Each pre‐processed EEG window $X_{t}$ is converted into a feature vector using the feature extraction function ϕ(·):

(6) $$f_{t}=\phi \left(X_{t}\right),f_{t}\in R^{14},$$

where $f_{t}$ represents the 14‐dimensional EEG feature vector extracted from the window $X_{t}$, and ϕ(·) denotes the feature extraction function. The final feature set includes frequency‐band powers, spectral ratios, statistical measures, Hjorth parameters, and spectral entropy.

First, frequency‐band power features are calculated from the standard EEG bands: delta, theta, alpha, and beta. For a frequency band b, the band power is computed as

(7) $$P_{b}\left(t\right)=\sum_{f=f_{b}^{low}}^{f_{b}^{high}}|\;S_{t}\left(f\right){|}^{2},$$

where $S_{t}\left(f\right)$ is the spectral representation of the EEG window $X_{t}$. Here, $f_{b}^{low}$ and $f_{b}^{high}$ indicate the lower and upper frequency limits of band b. These features show how the EEG signal energy is distributed across different frequency ranges.

Second, spectral ratio features are used to describe the relative balance between two EEG frequency bands. A general ratio feature is defined as

(8) $$R_{b_{1}/b_{2}}\left(t\right)=\frac{P_{b_{1}}\left(t\right)}{P_{b_{2}}\left(t\right)+\varepsilon },$$

where $P_{b_{1}}\left(t\right)$ and $P_{b_{2}}\left(t\right)$ are the band‐power values of two selected EEG bands. The small constant ε is added to avoid division by zero. In this work, theta/alpha and beta/alpha ratios are included to capture relative changes between important EEG rhythms.

Third, complexity‐related features are extracted to describe the irregular and dynamic nature of the EEG signal. A general complexity measure is represented as

(9) $$\;H_{t}=H\left(X_{t}\right),$$

where $H_{t}$ denotes the complexity value obtained from the EEG window $X_{t}$, and H(·) represents the complexity calculation. Hjorth parameters and spectral entropy are included because they help describe signal variability, waveform behavior, and spectral disorder.

Overall, the feature extraction stage converts each 2‐second EEG window into a 14‐dimensional feature vector. This compact representation reduces the difficulty of handling raw EEG data and provides suitable input for the Stage‐1 Random Forest classifier. The same feature vectors are also used in the Stage‐2 SARSA refinement stage, where window‐level decisions are made more stable using classifier confidence and previous decision information.

### 2.4 Stage 1: Random Forest–Based PD/HC Classification

In the first stage, each EEG window is classified as either Parkinson's disease or healthy control using a supervised Random Forest classifier. The extracted EEG feature vector is given as input to the classifier, and the model produces an initial class prediction for that window. Random Forest was selected for this study because EEG features often show complex and nonlinear relationships. In such cases, a single decision model may not capture the signal pattern effectively. Random Forest handles this problem by combining the decisions of several decision trees, which helps improve prediction stability and reduces the chance of overfitting. Another reason for choosing Random Forest is its interpretability. In biomedical classification, the model should not only provide an output but should also remain reasonably understandable. Compared with many complex learning models, Random Forest offers a simpler and more reliable option for EEG‐based PD/HC classification.

The PD/HC classification problem is treated as a supervised learning task. The training data are represented as

(10) $$D_{\mathrm{train}}={\left\{\left(f_{t},y_{t}\right)\right\}}_{t=1}^{M},$$

where $f_{t}\in R^{14}$ denotes the 14‐dimensional feature vector extracted from the EEG window $X_{t}$, $y_{t}\in \left\{HC,PD\right\}$ denotes the corresponding class label, and M is the total number of training windows. The Random Forest model learns the mapping between the extracted EEG features and the PD/HC label as

(11) $$\;{\hat{y}}_{t}^{RF}=F_{RF}\;\left(f_{t}\right),{\hat{y}}_{t}^{RF}\in \left\{HC,PD\right\},$$

where $F_{RF}\left(\cdot \right)$ is the trained Random Forest classifier, and ${\hat{y}}_{t}^{RF}$ is the initial class prediction for the *t*th EEG window. Along with this predicted label, the classifier also estimates the posterior probability of the Parkinson's disease class:

(12) $$p_{t}=P_{RF}\;\left(PD|f_{t}\right),$$

where $p_{t}$ represents the classifier confidence score for the PD class. This probability is used in the next stage to form the discretized confidence bin required for SARSA‐based temporal refinement.

Therefore, the Stage‐1 output for each EEG window consists of an initial PD/HC prediction and its corresponding confidence score. These outputs are not interpreted as cognitive‐state labels or stimulation‐control commands. They are used only by the SARSA stage to improve the stability of window‐level predictions across time.

In this study, the SARSA module has a limited and clearly defined role. The dataset used for evaluation does not provide DBS stimulation settings, cognitive task results, or measured responses to stimulation. Hence, SARSA is not used for selecting real DBS stimulation levels. Instead, the SARSA action space is defined using refined Parkinson's disease and healthy‐control decision labels. In the proposed framework, SARSA works as a temporal refinement step that improves the consistency of window‐level EEG predictions. It observes the decision pattern across neighboring EEG windows and helps reduce sudden label changes. Therefore, the SARSA component should be interpreted only as an offline decision‐refinement method for EEG‐based PD detection, not as a clinically validated closed‐loop DBS control system.

### 2.5 Stage 2: SARSA‐Based Temporal Decision Refinement

In the second stage of the proposed framework, SARSA is used to refine the sequence of decisions obtained from the Random Forest classifier. The Random Forest model first provides a window‐level prediction as either Parkinson's disease or a healthy control, along with its confidence value. SARSA then uses this confidence information and the previous decision pattern to improve the stability of predictions across nearby EEG windows. In this study, SARSA is not used to choose DBS stimulation levels and is not treated as a stimulation‐control method. Its purpose is only to improve the consistency of PD/HC classification in an offline resting‐state EEG analysis.

Unlike the Stage‐1 Random Forest classifier, which gives a separate prediction for each EEG window, SARSA considers the order of predictions over time. This is useful for resting‐state EEG analysis because nearby EEG windows can sometimes show sudden class changes due to noise, signal variation, or differences between subjects. In this study, SARSA is applied only to the Random Forest prediction sequence and not directly to the raw EEG signal. The SARSA state is built using two details: the classifier confidence score and the refined decision from the previous EEG window. This design helps reduce sudden changes in prediction and keeps the decision sequence more consistent across consecutive EEG windows. The refinement step also remains simple and easy to understand, instead of becoming another complicated black‐box process.

#### 2.5.1 SARSA Formulation for Temporal Label Refinement

In this study, SARSA is used only for temporal label refinement in offline EEG‐based PD/HC classification. Each EEG window is treated as one step in the ordered prediction sequence. First, the Random Forest classifier gives the initial PD/HC label for the current EEG window along with its confidence score. SARSA then uses this confidence value and the previously refined decision to adjust the current prediction more carefully. This helps avoid unnecessary label changes between neighboring EEG windows and produces a smoother, more stable decision sequence. In this framework, SARSA is not used as a separate diagnostic model or clinical control method. Its role is limited to refining the Random Forest prediction sequence.

For clarity, the notation used in the SARSA stage is defined before describing the state, action, reward, and update rule. Here, $W_{t}$ represents the EEG window at time index t, and $x_{t}$ represents the feature vector extracted from that window. The initial prediction given by the Random Forest classifier is denoted as ${\hat{y}}_{t}^{RF}$, while $p_{t}$ denotes the posterior probability of the Parkinson's disease class. This probability is converted into a discretized confidence bin, represented as $b_{t}$. The refined decision from the previous EEG window is denoted as $a_{t-1}$, and the current SARSA action $a_{t}$ represents the refined PD/HC label assigned to the present EEG window. Based on these terms, the SARSA state is written as $s_{t}=\left(b_{t},a_{t-1}\right)$. The same notation is followed consistently in the SARSA state, action, reward, and learning‐rule equations.

1. State

For each EEG window $X_{t}$, the Random Forest classifier provides the posterior probability of the Parkinson's disease class, denoted as $p_{t}$. This probability is converted into a discretized confidence bin. $b_{t}$. The SARSA state is defined as

(13) $$\;s_{t}=\left(b_{t},a_{t-1}\right),$$

where $b_{t}$ represents the confidence bin obtained from $p_{t}$, and $a_{t-1}$ represents the refined decision assigned to the previous EEG window. This state definition allows SARSA to use both the current classifier confidence and the recent prediction history.

1. Action

The SARSA action refers only to the refined class label assigned to the current EEG window. The action space is defined as

(14) $$a_{t}\in A=\left\{HC,PD\right\},$$

where HC denotes healthy control and PD denotes Parkinson's disease.

In the present study, SARSA is used in a very specific and limited manner. It does not work as a clinical decision system or as a DBS‐related control method. Its task is simply to refine the PD/HC label sequence already produced by the Random Forest classifier. The action space of SARSA contains only two refined labels: healthy control and Parkinson's disease. So, the action selected by SARSA should be read only as a corrected class label for the current EEG window. It does not represent any therapeutic action, stimulation level, DBS control setting, or cognitive‐state decision. This point is important because the dataset used here contains resting‐state EEG recordings only. It does not provide DBS settings, cognitive assessment scores, stimulation parameters, or stimulation‐response information.

1. Reward

The reward $r_{t}$ is used only during offline SARSA training. It is designed to support correct PD/HC classification and reduce unnecessary label switching between consecutive EEG windows. A positive reward is given when the refined decision matches the training label, while a penalty is given for an incorrect decision. A small switching penalty is also added when the refined label changes unnecessarily from one window to the next.

During testing, true labels are not used. The reward $r_{t}$ is not calculated, and no Q‐value update is performed. After training, the SARSA Q‐table is kept fixed and used only to refine the Random Forest prediction sequence for unseen test subjects. This training–testing separation avoids information leakage and clearly presents SARSA as an offline temporal decision‐refinement method.

#### 2.5.2 SARSA Learning

SARSA updates the action‐value function by using the present state–action pair and the next state–action pair selected by the same policy. In this study, this update is performed only during the offline training stage. The SARSA update rule is written as

(15) $$Q\left(s_{t},a_{t}\right)\leftarrow Q\left(s_{t},a_{t}\right)+\alpha \left[r_{t}+\gamma Q\left(s_{t+1},a_{t+1}\right)-Q\left(s_{t},a_{t}\right)\right],$$

where $Q(s_{t},a_{t})$ represents the action value for state $s_{t}$ and action $a_{t}$. Here, α∈(0,1) is the learning rate, γ∈(0,1) is the discount factor, $r_{t}$ is the reward used during training, and $s_{t+1}$ and $a_{t+1}$ denote the next state and next action selected by the current policy. Since SARSA is an on‐policy method, the Q‐value is updated using the action actually selected by the same policy.

SARSA is used in this work because it provides a stable way to refine window‐level prediction sequences. In resting‐state EEG analysis, neighboring windows may sometimes show sudden PD/HC label changes because of noise, signal variation, or subject‐level differences. The on‐policy nature of SARSA makes the refinement process more controlled than off‐policy methods such as Q‐learning. This is suitable for the present study because the aim is to improve prediction stability across consecutive EEG windows rather than produce frequent changes in the decision sequence.

During training, SARSA learns the refinement policy using the Random Forest confidence bin and the previous refined decision. After training, the learned Q‐table is fixed. During testing, the fixed SARSA policy is applied to predictions from unseen test subjects. At this stage, true labels are not used, rewards are not calculated, and Q‐values are not updated. Therefore, Stage 2 is used only as an offline temporal decision‐refinement stage for EEG‐based PD/HC classification.

**FIGURE 2:**
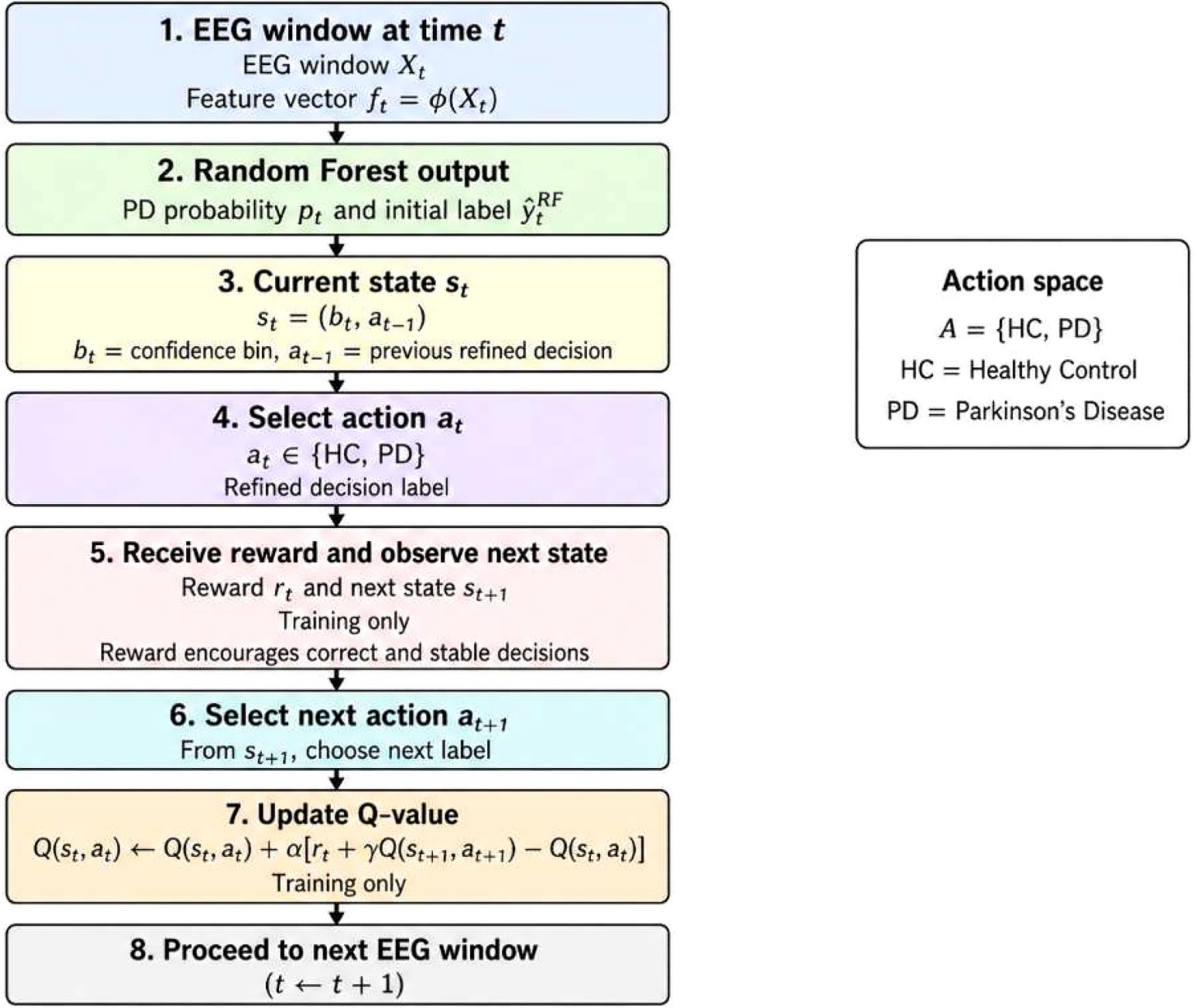
**SARSA‐based temporal decision refinement for resting‐state EEG‐based Parkinson's disease detection**.

Figure 2 shows the SARSA refinement steps used in the proposed method. For each EEG window $X_{t}$, the feature vector $f_{t}$ is extracted and passed to the Random Forest classifier. The classifier gives the PD probability $p_{t}$ and the initial prediction ${\hat{y}}_{t}^{RF}$. The probability $p_{t}$ is converted into a confidence bin $b_{t}$, and this value is combined with the previous refined decision $a_{t-1}$ to form the SARSA state $s_{t}=\left(b_{t},a_{t-1}\right)$. SARSA then selects the refined label $a_{t}$, which can be either HC or PD. During training, the reward $r_{t}$, next state $s_{t+1}$, and next action $a_{t+1}$ are used to update the Q‐value. During testing, no reward calculation or Q‐value update is performed. The trained SARSA policy is used only to make the prediction sequence more stable across consecutive EEG windows.

#### 2.5.3 Hybrid Integration of Random Forest and SARSA

The proposed framework uses Random Forest and SARSA in two connected stages for resting‐state EEG‐based Parkinson's disease detection. In the first stage, the Random Forest classifier analyses the extracted EEG features and gives window‐level predictions for Parkinson's disease and healthy‐control classes. In the second stage, SARSA improves the sequence of these predictions by considering the classifier confidence and the decision made in the previous EEG window. This step helps reduce sudden and inconsistent label changes, which may occur because resting‐state EEG signals are noisy and naturally variable. In the present study, the Random Forest–SARSA framework is used only as an offline EEG classification and temporal decision‐refinement model. It is not presented as an adaptive DBS stimulation‐control system.

### 2.6 Offline Evaluation and Temporal Decision‐Refinement Strategy

Although the EEG windows are processed in a time‐ordered manner, the proposed work remains an offline classification study. Each cleaned resting‐state EEG window is considered as one decision point for identifying whether the signal pattern belongs to the Parkinson's disease group or the healthy‐control group. First, the Random Forest model gives the initial PD/HC label and the Parkinson's disease probability for that window. After this step, SARSA is used in a limited and specific way. It looks at the classifier confidence and the previously refined decision, and then helps reduce sudden changes in the prediction sequence. During training, SARSA learns this refinement behavior only from the training‐fold outputs, confidence scores, previous decisions, and training labels. During testing, the learned Q‐table is kept unchanged. The test labels are not used, rewards are not calculated, and no Q‐value update is carried out. This separation is important because it avoids information leakage and gives a fairer estimate of performance on unseen subjects. Therefore, the framework should be understood only as an offline EEG‐based PD/HC classification and temporal decision‐refinement method.

For the *t*th EEG window, the offline decision‐refinement flow can be written as

(16) $$X_{t}\overset{\phi \left(\cdot \right)}{\to }f_{t}\overset{F_{RF}\left(\cdot \right)}{\to }\left({\hat{y}}_{t}^{RF},p_{t}\right)\to b_{t}\to \;s_{t}=\left(b_{t},a_{t-1}\right)\overset{\pi_{SARSA}}{\to }a_{t}$$

where $X_{t}$ is the preprocessed EEG window, ϕ(·) is the feature extraction function, $f_{t}$ is the extracted feature vector, $F_{RF}\left(\cdot \right)$ is the trained Random Forest classifier, ${\hat{y}}_{t}^{RF}$ is the initial RF prediction, and $p_{t}$ is the PD probability. The term $b_{t}$ represents the discretized confidence bin obtained from $p_{t}$, $s_{t}=\left(b_{t},a_{t-1}\right)$ is the SARSA state, $\pi_{SARSA}$ is the learned temporal refinement policy, and $a_{t}\in \left\{HC,PD\right\}$ is the refined label for the current EEG window.

During offline training, the SARSA interaction can be summarized as

(17) $$s_{t}\to a_{t}\to r_{t}\to s_{t+1},$$

Here, $r_{t}$ is the reward used only during training to support correct classification and reduce unnecessary label switching. During testing, rewards are not calculated, and Q‐values are not updated. The trained SARSA policy is kept fixed and is used only to refine the Random Forest prediction sequence for unseen test subjects. Therefore, this formulation represents offline temporal decision refinement for EEG‐based PD/HC classification, not DBS stimulation control or closed‐loop neuromodulation.

For evaluation, the framework adopts subject‐wise cross‐validation to ensure patient‐independent assessment and to avoid data leakage between training and testing subjects. Stage 1 performance is quantified using standard classification metrics, including accuracy, precision, recall, F1‐score, and area under the ROC curve (AUROC), reflecting the reliability of PD/HC classification. In addition, Stage 2 was assessed using policy stability, convergence behavior, and the consistency of refined PD/HC decisions across consecutive EEG windows. This evaluation supports the assessment of classification performance and temporal decision stability in the proposed offline framework.

The main methodological contributions of this study are summarized as follows:

- A subject‐independent resting‐state EEG pipeline is developed for PD/HC classification and benchmarking.
- A compact and interpretable EEG feature representation is used to support Random Forest–based classification.
- A SARSA‐based temporal refinement mechanism is introduced to make window‐level PD/HC predictions more consistent over time.
- A leakage‐free subject‐wise evaluation strategy is followed to assess both classification performance and temporal decision stability in a reproducible manner.

Overall, the novelty of this work lies in combining interpretable EEG feature‐based PD/HC classification with SARSA‐based temporal refinement in a lightweight offline framework. This approach provides a practical baseline for future EEG‐based Parkinson's disease detection studies, particularly when stable predictions across consecutive EEG windows are important.

Algorithm 1 **Hybrid RF +** **SARSA Framework for EEG‐Based PD/HC Classification**

Input: EEG recordings, window length $T_{w}$, feature extractor ϕ(·), action set A={HC,PD}

Output: Final subject‐level PD/HC prediction

1. Split the dataset using subject‐wise validation.
2. Preprocess EEG recordings and segment them into windows $X_{t}$.
3. Extract features from each window:
$$\;f_{t}=\phi \left(X_{t}\right)$$

Train the Random Forest classifier using training‐subject features only.

1. Obtain Random Forest prediction and PD probability $p_{t}$ for each window.
2. Form the SARSA state:
$$\;s_{t}=\left(b_{t},a_{t-1}\right),$$

1. Train SARSA using training labels and update the Q‐table.
2. Freeze the trained SARSA Q‐table.
3. Apply the trained Random Forest and frozen SARSA policy to the test subject.
4. Refine window‐level PD/HC labels using SARSA.
5. Combine refined window labels by majority voting.
6. Return the final subject‐level PD/HC prediction.

Where $b_{t}$ is the confidence bin from $p_{t}$.

## 3 Result and Discussion

### 3.1 Experimental Environment and Hardware Configuration

All experiments in this study were carried out in a carefully controlled computational environment to ensure methodological transparency, reproducibility, and fair performance evaluation of the proposed hybrid learning framework. The entire implementation was developed using Python (version 3.10 or later), supported by widely accepted scientific and machine‐learning libraries. Numerical computation and data handling were performed using NumPy and Pandas, (Harris et al. 2020, Pedregosa et al. 2011) signal processing operations were implemented with SciPy (Virtanen et al. 2020), and EEG‐specific preprocessing and data handling were conducted using MNE‐Python (Gramfort et al. 2013), which is a standard and well‐validated toolkit in EEG research. The machine‐learning modeling and evaluation procedures were performed using Scikit‐learn, which helped maintain consistency with commonly followed benchmarking practices. To ensure reproducibility, we have carefully listed the primary software used in this study and added them to the software list of the revised version, for example, NumPy, Pandas, SciPy, MNE‐Python, and Scikit‐learn.

For the reinforced learning component, we did not utilize an existing RL library. Instead, the SARSA component was implemented in Python. This facilitated a clear and detailed explanation of the feature representation, reward setting, and learning update function required for the Q‐value of the state–action pairs. Moreover, by explicitly detailing the SARSA component, we were able to clearly associate the EEG window with the SARSA learning step, i.e., the action. The computational cost of a single classical RL algorithm implementation is low compared with DNN‐based RL models.

Since one of the objectives of this work is to present a method that could be incorporated into standard machine‐learning research, we opted to implement the full method on a standard single‐workstation Intel‐class multicore platform with a minimum of 16 GB of RAM and enough free space for the BIDS‐formatted dataset.

Currently, community standards do not require the repository to include the raw data, but we understand that the reliance on shared data may change. We envision, though, that preprocessing and extraction of features could be scaled up as needed to perform on high‐performance resources. Yet, the full workflow with and without the SARSA component is efficient and reasonable to perform on standard hardware. The ordered windows of temporally sorted EEG windows were used and tested for stability with fixed ML and RL seeds.

### 3.2 Dataset Description and Characterization

The framework was tested with the publicly available OpenNeuro ds002778 resting‐state EEG dataset entitled UC San Diego Resting‐State EEG Data from Patients with Parkinson's Disease, which was accessed in version 1.0.5 with the DOI 10.18112/openneuro.ds002778.v1.0.5. The data follow the Brain Imaging Data Structure format, which provides a standard folder layout and clear metadata information. Since the dataset is publicly available and BIDS‐formatted, it allows for transparent analysis, reproducibility, and comparison with future studies. The dataset was cited as recommended in the OpenNeuro documentation (Rockhill et al. 2021) and references related to MNE‐BIDS and EEG‐BIDS were included to properly support the description of the dataset organization and EEG metadata structure (Appelhoff et al. 2019; Pernet et al. 2019).

The dataset comprises EEG recordings from two groups: Parkinson's disease subjects and healthy‐control participants, all obtained under resting‐state conditions, where the participants were not asked to perform any specific motor or cognitive task. This is a common condition to study, as it requires the least equipment and experimental setup, and resting‐state EEG is the easiest to record from participants, as it does not require them to perform any specific motor or cognitive task. Each subject provides one recording session of EEG data with the necessary physiological files and metadata to analyze the data.

The EEG signals were acquired at a uniform sampling rate, and the accompanying BIDS metadata describe key recording parameters such as channel configuration, reference information, and the local power‐line frequency (Appelhoff et al. 2019; Pernet et al. 2019). The recordings include both scalp EEG channels and auxiliary channels; however, for the present analysis, only scalp EEG channels were retained to ensure that extracted features represent true cortical activity. As expected in real clinical EEG, the dataset also exhibits natural intersubject variability due to differences in participant characteristics and disease‐related neural changes. This variability makes the classification task more challenging but also more meaningful for practical decision‐support research.

A key characteristic of resting‐state EEG is the absence of event markers or externally driven stimulus patterns. Therefore, the proposed framework must identify PD‐related differences using subtle changes in spontaneous brain rhythms and signal dynamics. For this reason, the analysis emphasizes interpretable spectral and statistical features derived from EEG windows, which aligns with clinically relevant EEG interpretation practice and supports explainable modeling.

The dataset has a moderate imbalance between the PD and HC groups, which is common in real biomedical datasets. The classes were not artificially balanced, so the evaluation reflects a more practical data condition. To obtain a fair estimate of model performance, subject‐wise validation was used. In this setup, all recordings from the same subject were kept within the same partition and were not shared between training and testing. This subject‐wise evaluation strategy helps minimize the risk of data leakage and gives a more realistic view of how the model performs when applied to unseen subjects.

Table 1 presents a small sample of the raw EEG values used in this study. The values are reported in microvolts and are taken from selected scalp EEG channels of both healthy‐control and Parkinson's disease samples. This table is included only to show the basic structure of the dataset and the general amplitude pattern observed in the resting‐state EEG recordings. It is not intended for direct statistical comparison between the PD and HC groups.

**TABLE 1:** Representative raw EEG signal values from the OpenNeuro ds002778 dataset for healthy‐control and Parkinson's disease samples.

Time (s)	EEG_Fp1 (µV)	EEG_Fp2 (µV)	EEG_C3 (µV)	EEG_C4 (µV)	EEG_Cz (µV)	Label
0.0	−18,559.01	−10,133.62	−14,554.77	−10,680.87	−9,909.15	HC
1.0	−18,546.61	−10,120.81	−14,587.49	−10,684.18	−9,898.25	HC
2.0	−18,432.95	−10,003.47	−14,606.58	−10,714.87	−9,895.50	HC
3.0	−18,527.33	−10,102.50	−14,605.43	−10,718.71	−9,876.93	HC
4.0	−18,544.79	−10,081.09	−14,639.08	−10,730.31	−9,858.28	HC
0.0	−20,397.51	−15,682.33	−1,492.51	−3,136.35	−3,463.70	PD
1.0	−20,384.17	−15,663.02	−1,485.83	−3,106.92	−3,446.04	PD
2.0	−20,390.51	−15,668.11	−1,482.67	−3,106.98	−3,457.88	PD
3.0	−20,389.57	−15,660.08	−1,482.58	−3,068.73	−3,440.98	PD
4.0	−20,386.76	−15,662.99	−1,484.67	−3,052.88	−3,474.85	PD

### 3.3 Preprocessing Methodology

EEG recordings usually contain several unwanted signal components. Baseline drift, power‐line interference, muscle‐related artifacts, and recording noise are common problems in raw EEG data. When these disturbances are not properly handled, they may affect the quality of the extracted features and reduce the reliability of the classification results. For this reason, all EEG recordings were processed through a structured preprocessing pipeline before feature extraction and model training. The main aim of this step was to reduce unwanted noise while preserving the EEG patterns that may be useful for Parkinson's disease analysis.

Table 2 shows the preprocessing steps used in this study. The procedure began with unit conversion, followed by scalp EEG channel selection, baseline correction, band‐pass filtering, notch filtering, common average referencing, normalization, windowing, and amplitude‐based rejection. After filtering, the EEG data were segmented into 2‐second windows without overlap. Windows presenting unusually high amplitude changes were identified and excluded using an amplitude peak‐to‐peak rejection criterion. The same preprocessing steps were used for all subjects to ensure that the PD and HC samples would be compared fairly with the Random Forest classifier and the SARSA algorithm in the subsequent analysis.

**TABLE 2:** Preprocessing steps and parameter settings used for resting‐state EEG analysis.

No.	Preprocessing step	Parameter/setting	Rationale
1	Signal unit conversion	Volts → microvolts (µV)	Improves numerical stability and interpretability
2	Channel selection	Scalp EEG channels only	Excludes auxiliary and non‐cortical signals
3	DC offset removal	Channel‐wise mean subtraction	Removes baseline drift
4	Band‐pass filtering	0.5–45 Hz (fourth‐order Butterworth)	Retains physiologically relevant EEG rhythms
5	Notch filtering	Power‐line frequency (50 Hz, metadata‐driven)	Suppresses electrical interference
6	Re‐referencing	Common Average Reference (CAR)	Reduces reference‐dependent bias
7	Signal normalization	Z‐score normalization (per channel)	Standardizes amplitude variability
8	Window segmentation	2‐second fixed‐length windows	Enables localized temporal analysis
9	Window overlap	No overlap	Simplifies temporal modeling
10	Artifact rejection	Amplitude thresholding	Removes windows with excessive noise

To maintain the reproducibility of the results, the same preprocessing steps were repeated in each validation fold. The raw EEG data were first converted to microvolts, and the auxiliary and non‐EEG channels were discarded. Next, the DC offset and slow base variation were reduced by subtracting the channel‐wise mean. The data were band‐pass filtered with a fourth‐order filter with cutoff frequencies of 0.5  and 45 Hz after the band‐pass transformation. A notch filter was applied at 50 Hz based on metadata. The signals were then common‐average‐referenced to reduce the impact of the reference. Z‐score normalization was performed in a leak‐free way. This was done for each validation fold by calculated the mean and standard deviation of the cleaned training data and applying the calculated values to the corresponding test set.

After preprocessing, the cleaned EEG signals were divided into 2‐second windows without overlap. The 150 µV artifact‐rejection threshold was applied to the peak‐to‐peak amplitude of these cleaned windows, not to the absolute raw values shown in Table 1. This distinction is important because Table 1 presents raw channel amplitudes before DC offset removal and filtering, where large baseline shifts may naturally appear. Each cleaned window was checked channel by channel. If the peak‐to‐peak amplitude of any retained scalp EEG channel exceeded 150 µV, that window was removed. This step helped exclude windows affected by sudden noise or large transient artifacts while keeping the useful EEG activity needed for feature extraction.

**FIGURE 3:**
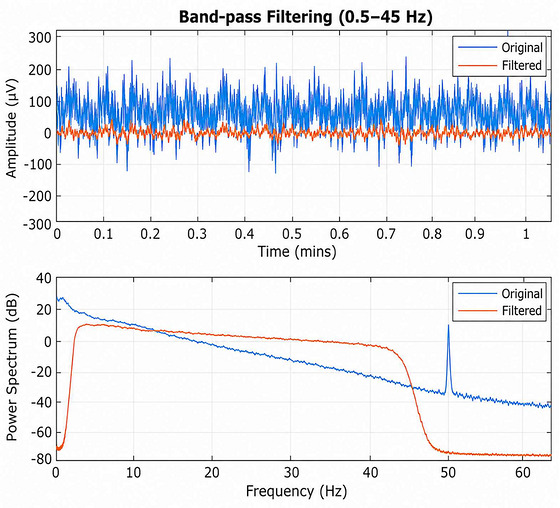
**Effect of 0.5–45 Hz band‐pass filtering on resting‐state EEG signals in the time and frequency domains**.

Figure 3 shows the EEG signal before and after preprocessing. In the time‐domain plot, the filtered signal appears smoother than the original signal, indicating that unwanted fluctuations were reduced. In the power‐spectrum plot, low‐frequency drift below 0.5 Hz and high‐frequency noise above 45 Hz are attenuated. The 50 Hz power‐line component is also reduced. This confirms that the filtering step preserved the useful EEG frequency range required for PD/HC classification.

After preprocessing, the cleaned EEG windows were passed to the feature extraction stage. This step helped improve the reliability of the subsequent Random Forest classification and SARSA‐based temporal refinement.

### 3.4 Feature Extraction Methodology

After preprocessing, the cleaned EEG signals were converted into meaningful features to describe patterns related to Parkinson's disease. Raw EEG data are not easy to use directly. They are lengthy, noisy, and non‐stationary, so giving the complete signal to a classifier may make the learning process difficult and less interpretable. For this reason, this study uses a compact feature set that captures the important spectral, statistical, and dynamic characteristics of resting‐state EEG activity.

The preprocessed EEG recordings were divided into fixed 2‐second windows, and features were extracted separately from each window. This window‐wise analysis helps the model observe short‐term changes in EEG activity instead of treating the whole recording as one continuous block. At the same time, the 2‐second window length provides sufficient information for spectral analysis. In this way, each EEG window is changed into a compact and interpretable feature representation before being used by the Random Forest classifier and the SARSA‐based temporal refinement stage.

For every window, features were first computed from the scalp EEG channels and then combined into a compact representation suitable for the Random Forest classifier and SARSA refinement stage (Jackson et al. 2019).

To cover different properties of the EEG signal, the extracted features were grouped into three categories: power‐domain features $\left(d_{p}\right)$, linear statistical features $\left(d_{l}\right)$, and complexity‐related features $\left(d_{c}\right)$. This grouping helps the model capture both basic EEG rhythm information and higher‐level signal variations without using complex deep‐learning representations.

Table 3 presents the final set of features used to describe each EEG window in a compact and interpretable way. The power‐domain features $\left(d_{p}\right)$ describe how signal energy is distributed across the main EEG frequency bands. The linear statistical features $\left(d_{l}\right)$ capture amplitude‐related properties such as signal strength and distribution pattern. The complexity‐related features $\left(d_{c}\right)$ describe signal irregularity and dynamic behavior. Together, these feature groups provide useful and complementary information for separating Parkinson's disease EEG patterns from healthy‐control EEG patterns in a computationally efficient manner.

**TABLE 3:** EEG feature set extracted from preprocessed resting‐state signals.

No.	Feature group	Feature name	Description
1	$d_{p}$	Delta band power (0.5–4 Hz)	Measures low‐frequency EEG activity
2	$d_{p}$	Theta band power (4–8 Hz)	Reflects cognitive and attentional processes
3	$d_{p}$	Alpha band power (8–13 Hz)	Associated with resting‐state rhythms
4	$d_{p}$	Beta band power (13–30 Hz)	Relevant for motor‐related neural activity
5	$d_{l}$	Theta:Alpha ratio	Captures relative spectral balance
6	$d_{l}$	Beta:Alpha ratio	Highlights motor‐related spectral changes
7	$d_{l}$	Root mean square (RMS)	Represents signal energy
8	$d_{l}$	Skewness	Quantifies signal asymmetry
9	$d_{l}$	Kurtosis	Measures signal peakedness
10	$d_{c}$	Hjorth activity	Represents signal variance
11	$d_{c}$	Hjorth mobility	Reflects signal frequency content
12	$d_{c}$	Hjorth complexity	Indicates waveform irregularity
13	$d_{c}$	Spectral entropy	Measures spectral disorder
14	$d_{c}$	Total band power	Summarizes overall EEG energy

Feature extraction was performed separately for every valid 2‐second EEG window. Spectral features were extracted from each valid 2‐second EEG window using Welch's power spectral density estimation. A Hamming window was used to obtain a stable frequency‐domain representation. The Welch segment length was fixed at 1 s, and adjacent segments were analyzed with 50% overlap. For a sampling frequency $f_{s}$, this setting corresponds to $nperseg=f_{s}$ and $noverlap=f_{s}\;/2$, giving an approximate frequency resolution of 1 Hz. The band‐power values were then calculated by integrating the PSD within the standard EEG frequency bands: delta, 0.5–4 Hz; theta, 4–8 Hz; alpha, 8–13 Hz; and beta, 13–30 Hz. The same PSD settings were followed for all subjects and validation folds to keep the feature extraction process consistent. Band‐power values were calculated from the standard EEG frequency bands, namely, delta, theta, alpha, and beta. From these band powers, theta/alpha and beta/alpha ratios were derived to observe relative changes between important EEG rhythms.

Along with the spectral features, time‐domain measures were also extracted. Root mean square was used to describe signal energy, while skewness and kurtosis helped represent the shape of the amplitude distribution. Hjorth activity, mobility, and complexity were calculated to capture signal variability and waveform behavior. Spectral entropy was also included to measure how the signal power was distributed across different frequency components.

All features were first computed at the channel level. The values were then averaged across the retained scalp EEG channels to form one compact feature vector for each EEG window. This channel‐averaging step reduced the feature dimension and made the representation less dependent on a specific electrode arrangement. Finally, each EEG window was represented using a 14‐dimensional feature vector, which was used for Random Forest classification and SARSA‐based temporal refinement.

Table 4 summarizes the preprocessing and feature extraction steps, outlining the parameters used in this process. We ensured an identical feature extraction procedure during each of the validation folds to obtain valid and reliable results. The artifact‐rejection threshold was applied only after preprocessing using the peak‐to‐peak amplitude of cleaned 2‐second EEG windows. This step was possible only after applying the band‐pass filter and removing the DC offset. This point is important to emphasize, as the raw values presented in Table 1 can have a large baseline shift before filtering and offset removal. We report the Welch PSD settings in Table 4, including window type, segment length, overlap, frequency resolution, and the EEG frequency ranges so that this process is fully transparent and can be replicated in future works. This includes the settings for calculating the power spectral density in Welch's method.

**TABLE 4:** Reproducibility summary of EEG preprocessing and feature extraction settings.

Processing component	Parameter/reporting detail
Dataset format	BIDS‐formatted OpenNeuro ds002778 resting‐state EEG dataset
Channel selection	Only scalp EEG channels were retained; auxiliary and non‐EEG channels were removed
Unit conversion	EEG amplitudes were converted from volts to microvolts
DC offset removal	Channel‐wise mean subtraction was applied
Band‐pass filtering	0.5–45 Hz, fourth‐order band‐pass filter
Notch filtering	50 Hz notch filter based on dataset metadata
Re‐referencing	Common average reference
Normalization	Z‐score normalization fitted on training subjects only and applied to the corresponding test subject
Window length	2 s
Window overlap	No overlap
Artifact rejection	150 µV peak‐to‐peak threshold applied to cleaned 2‐second windows; a window was rejected if any retained scalp channel exceeded this limit
Spectral estimation	Welch power spectral density estimation
Welch window type	Hamming window
Welch segment length	1 s
Welch overlap	50% overlap between adjacent segments
Frequency resolution	Approximately 1 Hz
EEG frequency bands	Delta: 0.5–4 Hz; theta: 4–8 Hz; alpha: 8–13 Hz; beta: 13–30 Hz
Feature groups	Band powers, spectral ratios, statistical measures, Hjorth parameters, and spectral entropy
Channel handling	Features were first computed at the channel level and then averaged across retained scalp channels
Final feature dimension	14 features per EEG window

### 3.5 Stage‐1 Random Forest Classification Results

After feature extraction, the first stage of the proposed hybrid framework employs a Random Forest (RF) classifier to estimate the cognitive state based on window‐level EEG features. Random Forest was selected due to its robustness to feature correlation, ability to handle nonlinear decision boundaries, and strong generalization performance with limited parameter tuning. These characteristics are particularly suitable for EEG data, where features often exhibit interdependencies and non‐stationary behavior.

The extracted feature vectors from all valid EEG windows were labeled according to subject class (Parkinson's disease or healthy control) and used to train the RF model. To prevent subject‐level data leakage, a subject‐wise validation strategy was followed. In this validation setup, all EEG windows belonging to a particular subject were kept within a single partition. In other words, data from the same subject were not allowed to appear in both the training and testing sets. This subject‐wise separation gives a more realistic estimate of how the model may perform when it is tested on completely unseen participants.

#### 3.5.1 Evaluation Protocol and Statistical Analysis

The model was evaluated using the same subject‐wise validation approach. This was done to keep the training and testing data clearly separated at the subject level. The OpenNeuro ds002778 dataset used in this work consisted of 31 participants, including 15 Parkinson's disease subjects and 16 healthy‐control subjects. As each participant produced multiple EEG windows, all windows from the same subject were placed in the same fold. This ensured that data from one subject did not appear in both the training and testing sets.

Leave‐one‐subject‐out validation was adopted because the dataset was small and the model needed to be tested on unseen participants. In each fold, one subject was used for testing, and the remaining subjects were used to train the Random Forest classifier and the SARSA refinement policy. The Random Forest model first produced a PD/HC prediction for each EEG window, along with a confidence score for that prediction. These outputs were then given to the trained SARSA policy. SARSA refined the window‐level decision sequence by considering the current confidence score and the previously refined decision. After this step, the refined predictions from all valid EEG windows of each participant were combined using majority voting to obtain one final subject‐level prediction.

The evaluation was reported at two levels for better clarity. The first level focused on fold‐aggregated window‐level performance, using the held‐out EEG windows from the leave‐one‐subject‐out validation folds. This result shows how well the model classified individual EEG windows and how SARSA improved the stability of consecutive PD/HC decisions. The second level focused on subject‐level performance. For this, all refined window predictions from each participant were combined through majority voting. Since the dataset contained 31 participants, the subject‐level analysis was based on 31 final PD/HC decisions. Based on this separation, the subject‐level confusion matrix, 95% confidence intervals, and paired comparison between Random Forest and RF + SARSA were reported separately.

For subject‐level evaluation, the final performance measures were calculated after majority‐vote aggregation. In this step, all valid window‐level predictions from each participant were combined to produce one final PD/HC decision. Since the dataset contained 31 participants, the subject‐level analysis was based on 31 final predictions. Accuracy, precision, recall, F1‐score, Macro‐F1, and AUROC were then calculated from these subject‐level decisions. The 95% confidence interval for accuracy was estimated using the subject‐level prediction results. The paired difference between the Stage‐1 Random Forest model and the final RF + SARSA model was tested using McNemar's exact test. This reporting method gives a clearer view of final participant‐level performance on unseen subjects.

For each subject, the final class label was obtained by combining the refined labels from all valid EEG windows of that subject. Since a single subject may contain many EEG windows, majority voting was used to decide the final subject‐level prediction. The aggregation rule is written as

(18) $$\;{\hat{Y}}_{i}=\mathrm{mode}\left(a_{i,1},a_{i,2},\text{…},a_{i,K_{i}}\right),$$

where ${\hat{Y}}_{i}$ represents the final predicted label for subject i, $a_{i,k}$ denotes the refined decision from the *k*th EEG window of that subject, and $K_{i}$ is the total number of valid EEG windows for subject i. This step ensures that the final decision is based on the overall window‐level pattern rather than a single EEG segment.

The 95% confidence interval was calculated as

(19) $$C\;I_{95\%}=\left[P_{2.5},P_{97.5}\right],$$

where $P_{2.5}$ and $P_{97.5}$ are the lower and upper percentile values obtained from the bootstrap distribution. This interval gives a clearer view of possible performance variation across subjects.

Table 5 summarizes the main evaluation settings used in this study. The OpenNeuro ds002778 dataset contained 31 subjects, including 15 Parkinson's disease subjects and 16 healthy controls. Leave‐one‐subject‐out validation was used so that the model could be tested on unseen subjects. To prevent data leakage, all EEG windows from the same subject were kept within the same fold. The Random Forest and SARSA models were trained only with training‐subject data. During testing, the SARSA Q‐table was kept fixed. Final results were reported at the subject level using majority voting, and 95% confidence intervals were estimated through subject‐level bootstrap resampling.

**TABLE 5:** Summary of the subject‐wise evaluation protocol.

Item	Revised reporting detail
Dataset	OpenNeuro ds002778
Total subjects	31
Class distribution	15 PD, 16 HC
Validation strategy	Leave‐one‐subject‐out validation
Leakage control	All windows from a subject kept in the same fold
Model training	RF and SARSA trained using training subjects only
Test‐time SARSA	Frozen Q‐table; no label reward or Q‐update
Metric level	Fold‐aggregated window‐level and subject‐level results reported separately
Window aggregation	Majority voting
Uncertainty estimate	95% CI using subject bootstrap
RF vs. RF + SARSA comparison	Paired subject‐level statistical comparison

#### 3.5.2 Classification Performance

The Stage‐1 Random Forest classifier was evaluated using accuracy, precision, recall, and F1‐score. These measures were selected because they provide a balanced view of model performance, especially when the Parkinson's disease and healthy‐control groups are not perfectly equal in size.

Accuracy shows the overall number of correct predictions. Precision indicates how reliable the positive predictions are, while recall shows how well the model identifies the target class. The F1‐score combines precision and recall into a single measure, which is useful when both false‐positive and false‐negative errors need to be considered.

All these metrics were calculated under the subject‐wise validation protocol. In this setup, EEG windows from the same participant were kept separate between training and testing. This helped reduce subject‐level data leakage and provided a more realistic estimate of Random Forest performance on unseen EEG data.

This helped avoid subject‐level data leakage, reduced overly optimistic results, and provided a more realistic estimate of how the Random Forest model performs on unseen resting‐state EEG data.

Table 6 compares the Stage‐1 Random Forest classifier with other baseline machine‐learning models. Random Forest gives better accuracy, F1‐score, Macro‐F1, and AUROC than SVM, k‐NN, and Decision Tree. This shows that Random Forest is a suitable first‐stage classifier for resting‐state EEG‐based Parkinson's disease detection. It provides a stable window‐level prediction before the SARSA‐based temporal refinement stage.

**TABLE 6:** Performance comparison of Stage‐1 machine‐learning classifiers for resting‐state EEG classification.

Method	Model type	Accuracy (%)	Precision (%)	Recall (%)	F1‐score (%)	Macro‐F1 (%)	AUROC
SVM (baseline)	ML	68.2	66.5	70.1	68.2	67.8	0.71
k‐NN	ML	65.9	64.2	66.8	65.5	65.0	0.69
Decision Tree	ML	66.7	65.8	67.5	66.6	66.1	0.70
Random Forest (Proposed)	ML	74.0	72.8	73.5	73.1	72.6	0.77

### 3.6 Stage‐2 SARSA Design and Optimization

Although the Stage‐1 Random Forest classifier gives useful window‐level predictions, resting‐state EEG signals can still vary from one window to another. Because of this natural variability, independent window‐level decisions may sometimes become unstable across time. To reduce this problem, a second stage based on SARSA is added to refine the prediction sequence.

In this study, SARSA is not used to classify raw EEG again. Instead, it works as a temporal refinement layer on top of the Random Forest outputs. It uses the classifier confidence score and the previous refined decision to make the prediction sequence more stable across consecutive EEG windows.

#### 3.6.1 SARSA State, Action, and Reward Design

- **State Representation**

The SARSA state is designed to be simple and directly connected to the Random Forest output. For each EEG window at time t, the state $s_{t}$ is defined as

(20) $$s_{t}=\left(b_{t},a_{t-1}\right),$$

where $b_{t}$ denotes the discretized Random Forest posterior probability for the Parkinson's disease class at window t. In this work, the probability is divided into 10 uniform bins, $b_{t}\in \left\{0,1,\text{…},9\right\}$, to reduce the effect of small confidence fluctuations. The term $a_{t-1}$ denotes the refined label assigned to the previous EEG window. This state representation allows SARSA to use both the current classifier confidence and the recent decision history.

- **Action Space**

The SARSA action represents the refined class label for the current EEG window. The action space is defined as

(21) $$a_{t}\in \left\{HC,PD\right\},$$

where HC denotes healthy control and PD denotes Parkinson's disease. This action does not represent any stimulation level or clinical control command. It only represents the refined PD/HC decision for the current EEG window.

- **Reward Function**

The reward function is used only during offline SARSA training. It is designed to encourage correct classification and reduce unnecessary switching between consecutive window labels. The reward at time t is defined as

(22) $$\;r_{t}=\left\{\begin{array}{cc} +1, & \mathrm{if}\;a_{t}=y_{t}\\ -1, & \mathrm{if}\;a_{t}\neq y_{t} \end{array}\right.\;-\lambda \cdot 1\left[a_{t}\neq a_{t-1}\right],$$

where $y_{t}$ is the true training label, 1[·] is the indicator function, and λ=0.05 is the switching penalty. The first part rewards correct decisions and penalizes wrong decisions. The second part discourages unnecessary label changes between nearby EEG windows. This helps improve temporal stability in the refined PD/HC prediction sequence.

#### 3.6.2 SARSA Learning Rule and Parameter Settings

The optimization is carried out using SARSA, which is an on‐policy temporal‐difference learning method.

(23) $$Q\left(s_{t},a_{t}\right)\leftarrow Q\left(s_{t},a_{t}\right)+\alpha \left[r_{t}+\gamma Q\left(s_{t+1},a_{t+1}\right)-Q\left(s_{t},a_{t}\right)\right],$$

where

α is the learning rate,

γ is the discount factor,

$r_{t}$ is the immediate reward,

$\left(s_{t+1},a_{t+1}\right)$ is the next state–action pair.

**Implementation Parameters**

The SARSA agent was trained using the following settings:

- Learning rate (α): 0.15
- Discount factor (γ): 0.90
- Exploration rate (ε): 0.20 (ε‐greedy policy)
- Episodes per fold: 600
- Probability bins: 10.

These parameters were empirically selected to balance exploration and exploitation while ensuring stable convergence across validation folds.

To avoid information leakage, the SARSA training and testing steps were separated in every subject‐wise validation fold. The Random Forest model was first trained using only the training subjects. Its window‐level predictions and confidence scores from the same training subjects were then used to train the SARSA Q‐table with label‐based rewards. After this training stage, the learned Q‐table was kept fixed. For test subjects, the Random Forest model produced window‐level predictions and confidence values, and the fixed SARSA policy was applied only to refine the prediction sequence. During testing, true labels were not used, rewards were not calculated, and no Q‐value updates were made. Therefore, the reported RF + SARSA performance represents temporal refinement on unseen test data without label leakage.

Table 7 highlights the benefit of using an on‐policy SARSA strategy for refining window‐level EEG decisions. Compared with Q‐Learning and DQN, the proposed SARSA‐based hybrid model achieves higher accuracy and improved class‐balanced performance (Macro‐F1), indicating more stable and reliable temporal decision refinement. The higher AUROC further suggests better overall separability between Parkinson's disease and healthy‐control patterns under the adopted evaluation protocol. The graphical comparison of reinforcement‐learning‐based temporal optimization methods is provided in Supplementary Figure S3.

**TABLE 7:** Comparative performance of reinforcement learning‐based temporal optimization methods for EEG classification.

Method	Model type	Accuracy (%)	Precision (%)	Recall (%)	F1‐score (%)	Macro‐F1 (%)	AUROC
Q‐Learning (temporal RL)	RL	72.4	71.1	70.8	71.0	70.2	0.74
Deep Q‐Network (DQN)	DL‐RL	76.8	75.4	77.1	76.2	75.6	0.81
Proposed SARSA	RL	78.6	77.9	79.2	78.5	78.0	0.83

#### 3.6.3 Final Hybrid Performance Evaluation

After SARSA‐based temporal refinement, the final hybrid framework was assessed at both the window level and the subject level. The window‐level results were calculated by combining the held‐out EEG‐window predictions obtained from all leave‐one‐subject‐out validation folds. This analysis mainly shows how far SARSA helped in making consecutive EEG‐window decisions more stable. For the subject‐level analysis, all valid window predictions belonging to each participant were combined through majority voting, and one final PD/HC label was assigned to that participant. This separation is important, especially because the dataset contains only 31 participants. Therefore, window‐level accuracy and subject‐level accuracy cannot be read in the same way.

At the window level, the RF + SARSA framework performed better than the Stage‐1 Random Forest model. The increase in recall suggests that the refined model was able to identify Parkinson's disease‐related EEG windows more effectively. The improvement in Macro‐F1 also indicates that the model handled the PD and HC classes in a more balanced way. In practical terms, SARSA worked only as a temporal refinement layer. It was not used as a separate clinical decision‐making module. Its main role was to make the EEG‐window prediction sequence more stable after the initial Random Forest classification. It helped reduce isolated window‐level prediction errors and made the decision sequence more consistent across neighboring EEG windows. The final subject‐level results were therefore reported separately using majority‐vote aggregation, confidence intervals, and McNemar's exact test.

Table 8 shows the fold‐aggregated window‐level results obtained across the leave‐one‐subject‐out validation folds. The Stage‐1 Random Forest model reached 74.0% accuracy, while the final RF + SARSA framework improved the accuracy to 78.6%. This improvement indicates that SARSA‐based temporal refinement helped make the EEG‐window predictions more stable and consistent. Here, it is important to note that the 78.6% value was calculated from held‐out EEG‐window predictions. It is not a direct subject‐level accuracy from the 31 participants. For this reason, the final participant‐level results are reported separately after majority‐vote aggregation.

**TABLE 8:** Fold‐aggregated window‐level performance of the Stage‐1 Random Forest and final RF + SARSA framework.

Method	Accuracy (%)	Precision (%)	Recall (%)	F1‐score (%)	Macro‐F1 (%)	AUROC
Stage‐1 Random Forest	74.0	72.8	73.5	73.1	72.6	0.77
Stage‐2 RF + SARSA	78.6	77.9	79.2	78.5	78.0	0.83

Table 9 gives the subject‐level confusion matrix after applying majority‐vote aggregation. In this stage, the result is no longer counted window by window. Instead, each participant is represented by one final PD/HC decision, which makes the result easier to understand from a participant‐level point of view. The Stage‐1 Random Forest model correctly identified 23 out of 31 participants. After applying SARSA‐based temporal refinement, the RF + SARSA model correctly identified 24 out of 31 participants. The improvement is small, but it is meaningful in this dataset, as one additional Parkinson's disease participant was correctly classified after the refinement step. This result suggests that SARSA helped reduce instability in the window‐level predictions and supported a more consistent final decision.

**TABLE 9:** Subject‐level confusion matrix after majority‐vote aggregation.

Model	Actual/Predicted	Predicted HC	Predicted PD
Random Forest	Actual HC	12	4
Random Forest	Actual PD	4	11
RF + SARSA	Actual HC	12	4
RF + SARSA	Actual PD	3	12

Table 10 reports the final subject‐level performance after majority‐vote aggregation. Here, each participant is counted only once, so the results give a clearer view of final PD/HC classification at the participant level. The RF + SARSA model correctly classified 24 out of 31 participants, compared with 23 out of 31 participants by the Stage‐1 Random Forest model. This gives a subject‐level accuracy of 77.4% for RF + SARSA and 74.2% for Random Forest.

**TABLE 10:** Subject‐level performance with 95% confidence intervals and paired statistical comparison.

Model	Correct/Total	Accuracy (%)	Precision (%)	Recall (%)	F1‐score (%)	Macro‐F1 (%)	AUROC	95% CI for accuracy	Statistical comparison
Random Forest	23/31	74.2	73.3	73.3	73.3	74.2	0.77	56.8−86.3	Reference
RF + SARSA	24/31	77.4	75.0	80.0	77.4	77.4	0.81	60.2–88.6	McNemar's exact test, *p* = 1.000

Precision, recall, and F1‐score were calculated by taking the PD class as the positive class. Macro‐F1 was obtained by averaging the F1‐scores of both HC and PD classes. The 95% confidence interval was calculated from the 31 subject‐level predictions using the Wilson score method. McNemar's exact test was then applied to the paired subject‐level predictions of Random Forest and RF + SARSA. The obtained *p*‐value of 1.000 shows that the subject‐level improvement was not statistically significant. This is expected because the dataset contains only 31 participants. Therefore, the result should be understood as a small improvement in decision consistency, not as strong statistical evidence of clinical superiority.

The final RF + SARSA framework obtained 78.6% accuracy in the fold‐aggregated window‐level evaluation across the leave‐one‐subject‐out validation folds. This value was not considered as direct subject‐level accuracy, because the dataset included only 31 participants. For the subject‐level analysis, all valid window predictions from each participant were combined through majority voting, and one final PD/HC label was assigned to each participant. Based on these 31 final subject‐level decisions, the Stage‐1 Random Forest model correctly classified 23 participants, while the RF + SARSA model correctly classified 24 participants. This corresponds to subject‐level accuracies of 74.2% and 77.4%, respectively. The 95% confidence interval for the RF + SARSA subject‐level accuracy was 60.2%–88.6%. McNemar's exact test did not show a statistically significant difference between Random Forest and RF + SARSA at the subject level, with *p* = 1.000. This outcome is understandable, as the study used a small subject group. Therefore, the improvement should be viewed as a descriptive improvement in subject‐level decision consistency, rather than strong statistical proof of clinical superiority.

**FIGURE 4:**
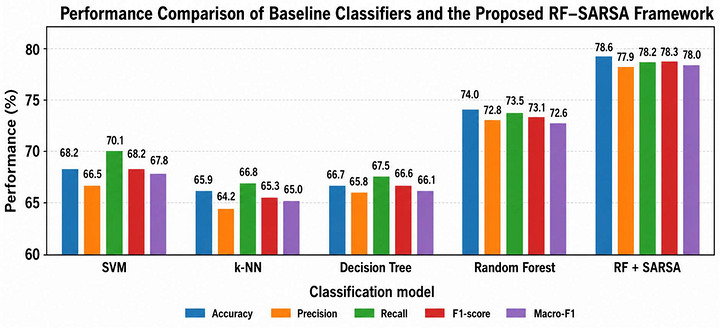
Performance comparison of baseline classifiers and the proposed RF + SARSA framework.

Figure 4 compares the baseline classifiers with the proposed RF + SARSA model for resting‐state EEG‐based Parkinson's disease detection. Among the individual machine‐learning models, Random Forest performs better than SVM, k‐NN, and Decision Tree in most of the reported metrics. After SARSA‐based temporal refinement is included, the final RF + SARSA model shows further improvement, especially in accuracy, recall, F1‐score, and Macro‐F1. This result indicates that SARSA reduces unstable window‐level predictions and supports a more consistent subject‐level PD/HC decision. Overall, the figure shows that the proposed RF + SARSA framework provides more balanced classification performance than the baseline models.

Table 11 presents the key parameter settings used in the proposed RF + SARSA model. The Random Forest parameters were selected to support stable window‐level learning, reduce feature‐related bias, and manage class imbalance. In the SARSA stage, a compact state representation was used by combining the classifier confidence score with the previously refined decision. The reward function also included a small switching penalty to prevent unnecessary changes in the predicted label from one EEG window to the next. With this setting, the model was able to maintain better temporal consistency and produce more stable subject‐level decisions without making the framework overly complex.

**TABLE 11:** Key parameter settings of the proposed RF + SARSA hybrid framework.

Module	Key parameters	Setting (summary)	Purpose
Stage‐1 Random Forest	Trees/split rule	n_estimators = 300, criterion = gini	Stable ensemble learning for EEG features
Stage‐1 Random Forest	Feature sampling	max_features = sqrt	Reduces correlation and improves generalization
Stage‐1 Random Forest	Regularization	max_depth = None, min_samples_split = 2, min_samples_leaf = 1	Controls tree growth and avoids overfitting
Stage‐1 Random Forest	Robust training	bootstrap = True, class_weight = balanced	Handles variance and class imbalance
Stage‐1 Random Forest	Reproducibility/evaluation	random_state = 42, subject‐wise split	Ensures repeatability and prevents leakage
Stage‐2 SARSA	State/actions	*s_t_* = (*b_t_*, *a_t_*_‐1_), actions = {HC, PD}, bins = 10	Encodes RF confidence and temporal context
Stage‐2 SARSA	Reward	±1 −λ1 [*a_t_​* ≠ *a_t_*_‐1_], λ = 0.05	Correctness + switching penalty (stability)
Stage‐2 SARSA	Learning	α = 0.15, episodes = 600, tabular (Q (s, a))γ=0.90ε=0.20,	On‐policy temporal optimization
Decision aggregation	Subject label rule	Majority vote/mean score	Converts window decisions to subject prediction

#### 3.6.4 Temporal Consistency Baseline Comparison

To examine the usefulness of SARSA‐based temporal refinement, the proposed method was compared with standard temporal smoothing techniques. This comparison was needed because SARSA works on a sequence of EEG‐window predictions, so its contribution should be checked against commonly used postprocessing methods.

The baseline methods used in this study were majority voting, hysteresis‐based thresholding, and HMM/Viterbi decoding. Majority voting assigns the final label based on the class predicted most often across EEG windows. Hysteresis thresholding reduces frequent label switching by allowing a class change only when the classifier confidence is strong enough. HMM/Viterbi decoding identifies the most likely label sequence by considering transition patterns between states. All these methods were evaluated using the same subject‐wise validation protocol to keep the comparison fair and consistent.

The results indicate that simple temporal smoothing methods can improve prediction stability when compared with the Stage‐1 Random Forest model. However, the RF + SARSA framework gives the strongest overall performance in terms of accuracy, Macro‐F1, AUROC, and temporal consistency. This improvement is mainly because SARSA uses both the classifier confidence and the previous decision context while learning a refinement policy from the training subjects. Majority voting and hysteresis use fixed rules, and HMM/Viterbi depends on predefined transition assumptions. These methods may not fully represent the natural variability present in resting‐state EEG signals. Therefore, SARSA gives a stronger and more flexible temporal refinement approach while still keeping the proposed model lightweight and interpretable.

Table 12 compares the proposed RF + SARSA framework with standard temporal refinement methods. The Stage‐1 Random Forest model, without any temporal refinement, achieved 74.0% accuracy and 0.77 AUROC. After applying simple temporal methods, the performance improved gradually. Majority voting increased the accuracy to 75.2%, hysteresis‐based label switching improved it to 76.4%, and HMM/Viterbi decoding reached 77.3%. The proposed RF + SARSA framework achieved the best overall result, with 78.6% accuracy, 78.5% F1‐score, 78.0% Macro‐F1, and 0.83 AUROC. This comparison shows that SARSA‐based temporal refinement gives better decision stability than fixed smoothing methods while keeping the model lightweight and interpretable.

**TABLE 12:** Performance comparison of temporal refinement methods for resting‐state EEG‐based Parkinson's disease detection.

Method	Temporal strategy	Accuracy (%)	F1‐score (%)	Macro‐F1 (%)	AUROC
Stage‐1 RF	No temporal refinement	74.0	73.1	72.6	0.77
RF + Majority Vote	Subject‐level aggregation only	75.2	74.8	74.3	0.78
RF + Hysteresis	Confidence‐based label switching	76.4	76.0	75.5	0.80
RF + HMM/Viterbi	Probabilistic sequence decoding	77.3	77.0	76.4	0.81
RF + SARSA	Learned temporal refinement	78.6	78.5	78.0	0.83

**FIGURE 5:**
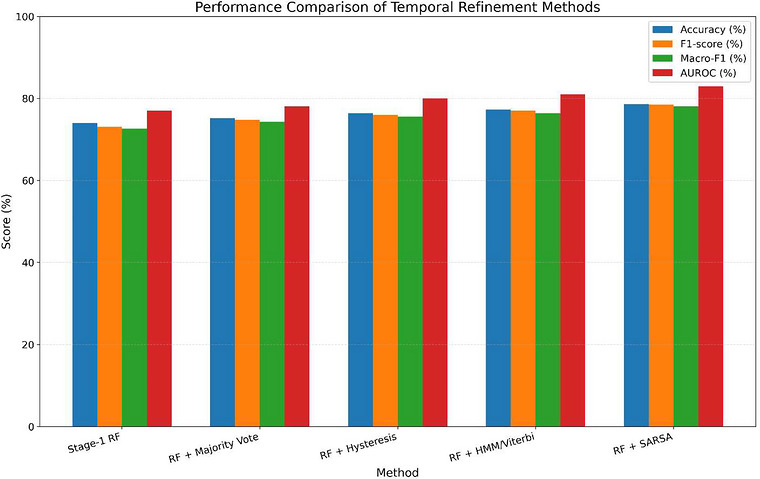
Performance comparison of temporal refinement methods for resting‐state EEG‐based Parkinson's disease detection.

Figure 5 compares the Stage‐1 Random Forest model with different temporal refinement methods using accuracy, F1‐score, Macro‐F1, and AUROC. The Stage‐1 Random Forest result is used as the baseline without temporal refinement. After the application of majority voting, hysteresis‐based label switching, and HMM/Viterbi decoding, the performance gradually improved. The proposed RF + SARSA framework obtained the best‐performing overall results with 78.6% accuracy, 78.5% F1‐score, 78.0% Macro‐F1, and 0.83 AUROC. This result indicates that SARSA‐based temporal refinement provides predictions that are less prone to change over time compared with a fixed smoothing method, while at the same time maintaining the advantage of low complexity and good interpretability of the model.

#### 3.6.5 Key Contributions Supported by Experimental Results

##### 3.6.5.1 Reproducible BIDS‐Compliant EEG Processing Pipeline

An open‐source BIDS‐aligned preprocessing workflow was used to convert the EEG readings into a uniform format. The steps for unit conversion, common average referencing, band‐pass filtering, notch filtering, z‐score normalizing, and windowing were included. Clean and robust EEG segments were obtained to evaluate the proposed approach in a subject‐independent scenario.

##### 3.6.5.2 Interpretable Multi‐Domain Feature Design

A small feature set was designed by combining spectral band powers, spectral ratios, statistical measures, Hjorth parameters, and spectral entropy, which characterize the capturing of rhythmic and random behavior in the resting‐state EEG readings, indicating the presence of useful information for PD/HC classification with proper interpretability for the feature representation.

##### 3.6.5.3 Leakage‐Free Stage‐1 Random Forest Baseline

The subject‐wise verified Stage‐1 Random Forest model provides an estimate of PD/HC classification performance on unseen subjects, keeping the model's predictive power and generalization ability unbiased by the validation procedure. At the same time, the model remained interpretable and computationally efficient.

##### 3.6.5.4 SARSA‐Based Temporal Refinement for Decision Stability

The Stage‐2 SARSA module refined the window‐level predictions by using the classifier confidence score and the previously refined decision. This reduced sudden label changes and improved temporal consistency across consecutive EEG windows.

##### 3.6.5.5 Lightweight Final Hybrid RF + SARSA Framework

The final RF + SARSA framework improved subject‐level performance without depending on heavy deep‐learning architectures. Therefore, the proposed method is more suitable for reproducible EEG‐based Parkinson's disease detection studies where simplicity, interpretability, and stable decision‐making are important.

### 3.7 Comparative Analysis

Recent EEG‐based Parkinson's disease classification studies commonly follow either feature‐based machine‐learning approaches or end‐to‐end deep‐learning approaches. Deep‐learning models may give good results in some cases, but their performance is often influenced by dataset size, preprocessing choices, and validation design. In addition, their internal decision process may be difficult to explain in biomedical applications. Feature‐based machine‐learning methods are generally simpler and more transparent. However, when EEG decisions are made window by window, these methods may not fully manage the natural variability present in resting‐state EEG signals. This limitation supports the need for a method that combines interpretable classification with temporal refinement. In contrast, classical ML methods are typically more transparent and computationally efficient, but they may struggle with the temporal variability and non‐stationary nature of resting‐state EEG when decisions are made window by window. The proposed framework addresses this practical gap by combining a clear Random Forest classifier with SARSA‐based temporal refinement. Random Forest gives the initial PD/HC prediction for each EEG window, while SARSA helps make the prediction sequence smoother by reducing unnecessary label changes. This is useful in resting‐state EEG analysis, where predictions may vary across windows because of signal noise and natural differences between subjects.

In this way, the method maintains a reasonable balance between performance, interpretability, and reproducibility. It does not depend on a heavy black‐box model, and it is evaluated using subject‐wise validation to provide a fairer understanding of its performance on unseen participants. Therefore, the proposed approach can be viewed as a practical offline EEG‐based PD/HC classification framework with improved temporal decision consistency.

Table 13 shows that the proposed RF + SARSA framework achieves stronger overall performance than the listed recent baselines, while also providing more balanced class‐wise behavior. The improvement is particularly meaningful because SARSA refines window‐level decisions by incorporating temporal consistency, reducing unstable prediction changes across consecutive EEG windows. The AUROC value also shows better separation between Parkinson's disease and healthy‐control classes, which supports the reliability of the proposed method under the selected evaluation protocol. A graphical comparison with selected public EEG‐based Parkinson's disease studies is included in Supplementary Figure S4.

**TABLE 13:** Comparative performance of recent EEG‐based Parkinson's disease classification approaches and the proposed hybrid RF + SARSA framework.

Study (year)	Approach	Model type	Accuracy (%)	Precision (%)	Recall (%)	F1‐score (%)	Macro‐F1 (%)	AUROC	DOI / ID
	CL‐Encoder + Freeze	DL	69.40	71.6	68.8	68.2	NR	0.656	arXiv:2408.00906
	Random Forest baseline	ML	69.0	74.0	56.0	64.0	NR	0.81	10.18293/DMSVIVA2025‐003
	CNN‐LSTM Hybrid (Hyb2)	DL	73.0	66.0	92.0	77.0	NR	0.79	10.18293/DMSVIVA2025‐003
	LSTM	DL	65.0	64.0	64.0	64.0	NR	0.72	10.18293/DMSVIVA2025‐003
Proposed	RF + SARSA	Hybrid (ML + RL)	78.6	77.9	79.2	78.5	78.0	0.83	—

### 3.8 Discussion

The results of this study show that resting‐state EEG can support Parkinson's disease classification when the analysis pipeline is carefully designed and consistently applied. This task is still challenging because resting EEG does not include task markers, and the signal naturally changes over time. Therefore, the model must handle subject‐to‐subject variation as well as short‐term signal changes within the same recording. The reported results, therefore, reflect a realistic scenario where models must generalize across subjects and remain robust to spontaneous neural fluctuations.

The preprocessing and feature design contributed directly to the stability of the learning process. Physiologically valid filtering, notch suppression, CAR re‐referencing and normalization helped reduce baseline drift and electrical interference, while fixed windowing enabled consistent feature extraction across recordings. The selected multi‐domain feature set—combining spectral power patterns with statistical and complexity measures—captures both rhythmic activity and irregular dynamics that are commonly altered in PD, while remaining interpretable for biomedical audiences.

At the modeling level, the Stage‐1 Random Forest classifier provides a transparent and computationally efficient baseline for window‐level state estimation under strict subject‐wise evaluation. However, window‐wise decisions alone may fluctuate in resting EEG due to short‐term variability and residual noise. This is the key motivation for introducing Stage‐2 SARSA refinement, which explicitly incorporates temporal context through classifier confidence bins and the previous decision, and rewards consistent behavior while penalizing unnecessary switching.

In this study, the SARSA module is used only as an offline temporal refinement method. The label‐based reward is applied during training to learn a policy that can make window‐level PD/HC decisions more stable. During testing, the trained policy is applied to unseen subjects without using true labels and without further Q‐value updates. This separation is necessary because using ground‐truth labels while testing would cause information leakage and may produce unrealistically high performance. To avoid this problem, the SARSA Q‐table is fixed after training and is used only to refine the Random Forest prediction sequence during test evaluation.

The results suggest that temporal refinement has a practical value in resting‐state EEG‐based PD/HC classification. The Stage‐1 Random Forest model gives a simple, interpretable, and computationally light baseline. However, as is usually seen in EEG analysis, window‐level predictions may not always remain stable, mainly because resting‐state EEG signals are noisy, non‐stationary, and vary from one subject to another. In this context, the SARSA stage adds a useful refinement layer. It uses the classifier confidence and the previously refined decision to control sudden label changes between neighboring EEG windows. This makes the prediction sequence more stable and supports a more reliable aggregation of window‐level outputs. At the same time, the findings must be understood within the correct scope. The present work is only an offline EEG‐based PD/HC classification and temporal decision‐refinement study. It does not make any claim related to cognitive‐state estimation, DBS control, or clinical neuromodulation.

## 4 Conclusion

This study presented a lightweight hybrid Random Forest–SARSA framework for offline Parkinson's disease and healthy‐control classification using resting‐state EEG signals. The framework was evaluated on the publicly available OpenNeuro ds002778 dataset through leave‐one‐subject‐out validation. This subject‐wise validation design ensured that all EEG windows from a given participant would appear in training and testing folds of the same model validation process. Such separation is necessary to minimize subject‐level data leakage and to obtain a realistic estimate of model performance in new, unseen participants.

The proposed method is designed in two stages. In the first stage, a Random Forest classifier employed a 14‐dimensional compact EEG feature set to obtain window‐level PD/HC predictions. In the 14‐dimensional feature set frequency‐band powers, spectral ratios, statistical measures, Hjorth parameters, and spectral entropy were included. In the second stage, offline temporal decision refinement using SARSA was applied. It used the confidence score of the classifier and the decision already refined in the previous window to minimize sudden, for example, frame‐by‐frame changing of labels in a local group of windows. This design of method kept the model simple, easily interpretable, and suitable for reproducible resting‐state EEG analysis.

The final RF + SARSA model obtained 78.6%‐fold‐aggregated window‐level accuracy, which is higher compared with 74.0% accuracy obtained by Stage‐1 Random Forest model. This result endorses the hypothesis that proposed SARSA‐based refinement improves the stability of window‐level EEG predictions. However, this value was not considered as direct subject‐level accuracy because the dataset contained only 31 participants. For subject‐level evaluation, all valid window‐level predictions of each participant were combined using majority voting. Based on the 31 final participant‐level decisions, the Random Forest model correctly classified 23 participants, whereas the RF + SARSA model correctly classified 24 participants. This corresponds to subject‐level accuracies of 74.2% and 77.4%, respectively. The 95% confidence interval for RF + SARSA subject‐level accuracy was 60.2%–88.6%, and McNemar's exact test did not show a statistically significant subject‐level difference between Random Forest and RF + SARSA, *p* = 1.000. Therefore, the improvement should be understood as a descriptive gain in subject‐level decision consistency rather than strong statistical evidence of clinical superiority.

The scope of this work is purposely limited to offline resting‐state EEG‐based PD/HC classification. The dataset does not contain cognitive assessment scores, DBS stimulation parameters, stimulation‐response measurements, medication‐state details, or longitudinal clinical outcome records. For this reason, SARSA should not be understood as a DBS controller, stimulation‐action selector, cognitive‐state estimator, or clinically validated neuromodulation policy. In this study, SARSA is used only as a temporal refinement step to make window‐level PD/HC predictions more consistent.

The findings are promising, but they should be interpreted with caution at this stage. Before making any clinical‐level claim, the RF + SARSA framework needs further validation using larger and independent EEG datasets. Future studies should also include multicenter data and more diverse patient groups. This would help show how well the model performs across different recording conditions, subject characteristics, and clinical settings. Including disease‐stage information, medication‐state details, longitudinal follow‐up data, and external validation cohorts would strengthen the clinical relevance of the model. Any future extension toward DBS‐related or real‐time clinical applications should be treated as a separate research direction and must be supported by proper stimulation parameters, physiological response data, and prospective clinical validation.

## Author Contributions

**Sanju S**: conceptualization, methodology, data curation, formal analysis, writing – original draft, writing – review and editing. **S. Edward Rajan**: visualization, supervision, validation.

## Funding Information

The authors have nothing to report.

## Conflicts of Interest

The authors declare no conflict of interest.

## Supporting information




**Supplementary materials**: brb371563‐sup‐0001‐SuppMat.docx

## Data Availability

The dataset used in this study is publicly available from OpenNeuro as ds002778, titled *UC San Diego Resting‐State EEG Data from Patients with Parkinson's Disease*, version 1.0.5, DOI: 10.18112/openneuro.ds002778.v1.0.5. The processed data and analysis details are available from the corresponding author upon reasonable request.
