# Supplementary material for: A Hybrid Random Forest–SARSA Framework for Resting‐State EEG‐Based Parkinson's Disease Detection With Temporal Decision Refinement
Source: Brain Behav. 2026 Jul 9;16(7):e71563. doi: 10.1002/brb3.71563 (PMC13347173; doi:10.1002/brb3.71563)
Supplement: Supplementary file 1 — Supplementary materials: brb371563‐sup‐0001‐SuppMat.docx [file BRB3-16-e71563-s001.docx]

**Supplementary Material**

**Supplementary Section S1. Additional Feature Analysis**


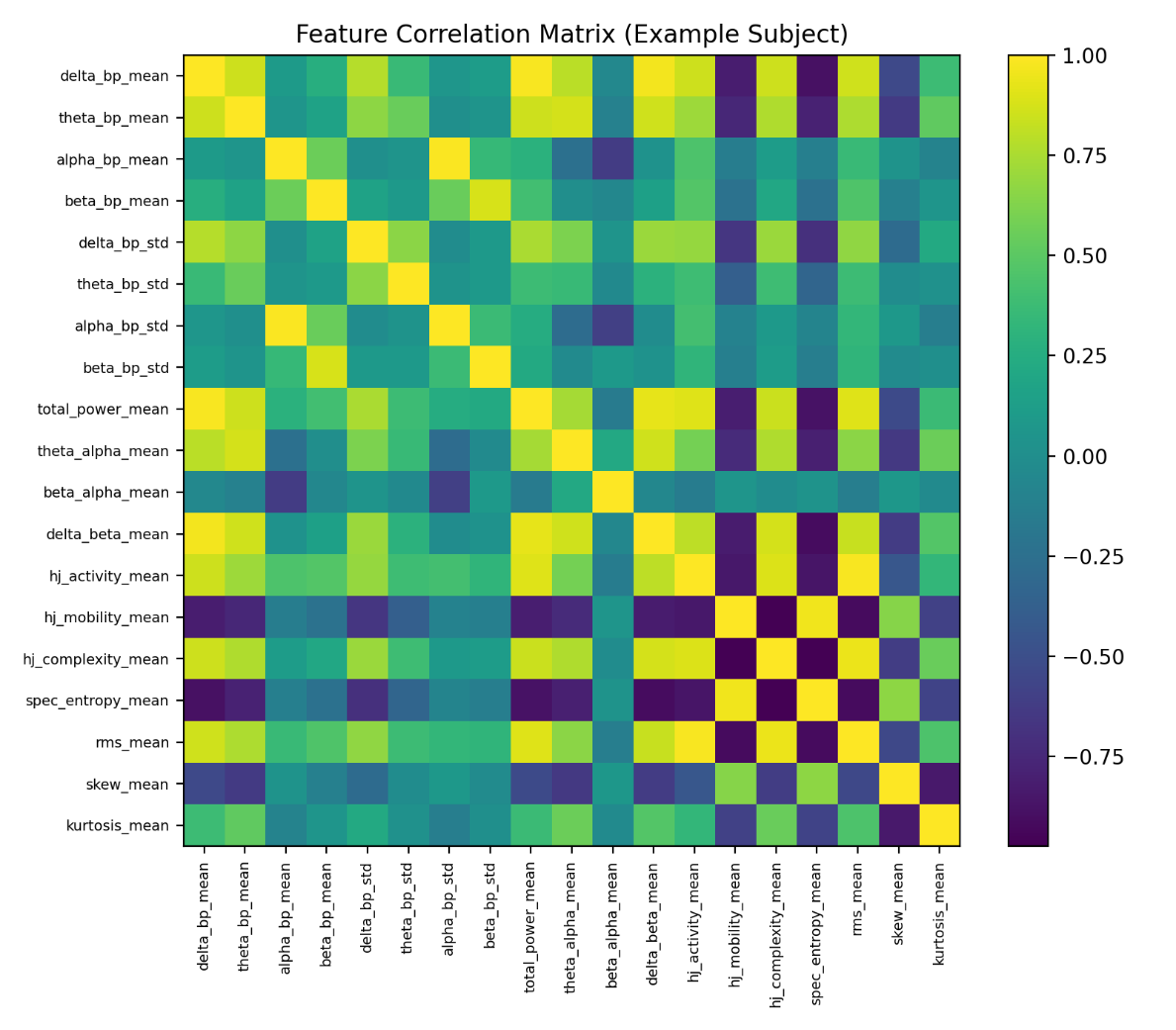


**Supplementary Figure S1. Feature correlation matrix for an example subject using the extracted EEG feature set.**

Supplementary Figure S1 shows the relationship among the extracted EEG features. Some features show positive or negative correlations, which is expected because band powers and ratio-based features may share related information. Other features, such as Hjorth parameters and spectral entropy, provide different signal characteristics and add complementary value. This figure supports the use of a mixed feature set by showing that the selected features are structured and informative for the Random Forest classifier and SARSA-based temporal refinement stage.

**
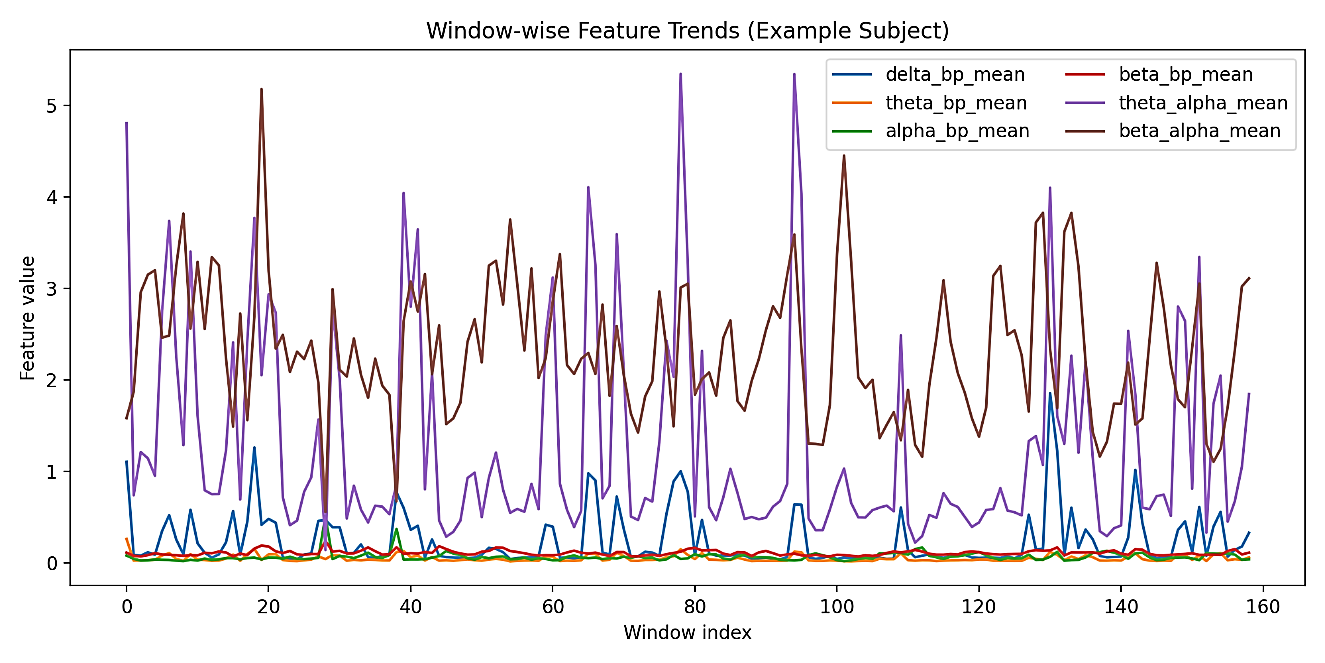
**

**Supplementary Figure S2. Window-wise variation of selected EEG features for an example subject.**

Supplementary Figure S2 shows how selected EEG features change across consecutive windows for one example subject. Such variation is expected in resting-state EEG because the signal is naturally non-stationary. The observed changes indicate that the extracted features capture useful spectral variations across time. This supports their use in the Random Forest classification stage and in the SARSA-based temporal refinement stage, where prediction stability across EEG windows is important.

**Supplementary Section S2. Additional Model Comparisons**


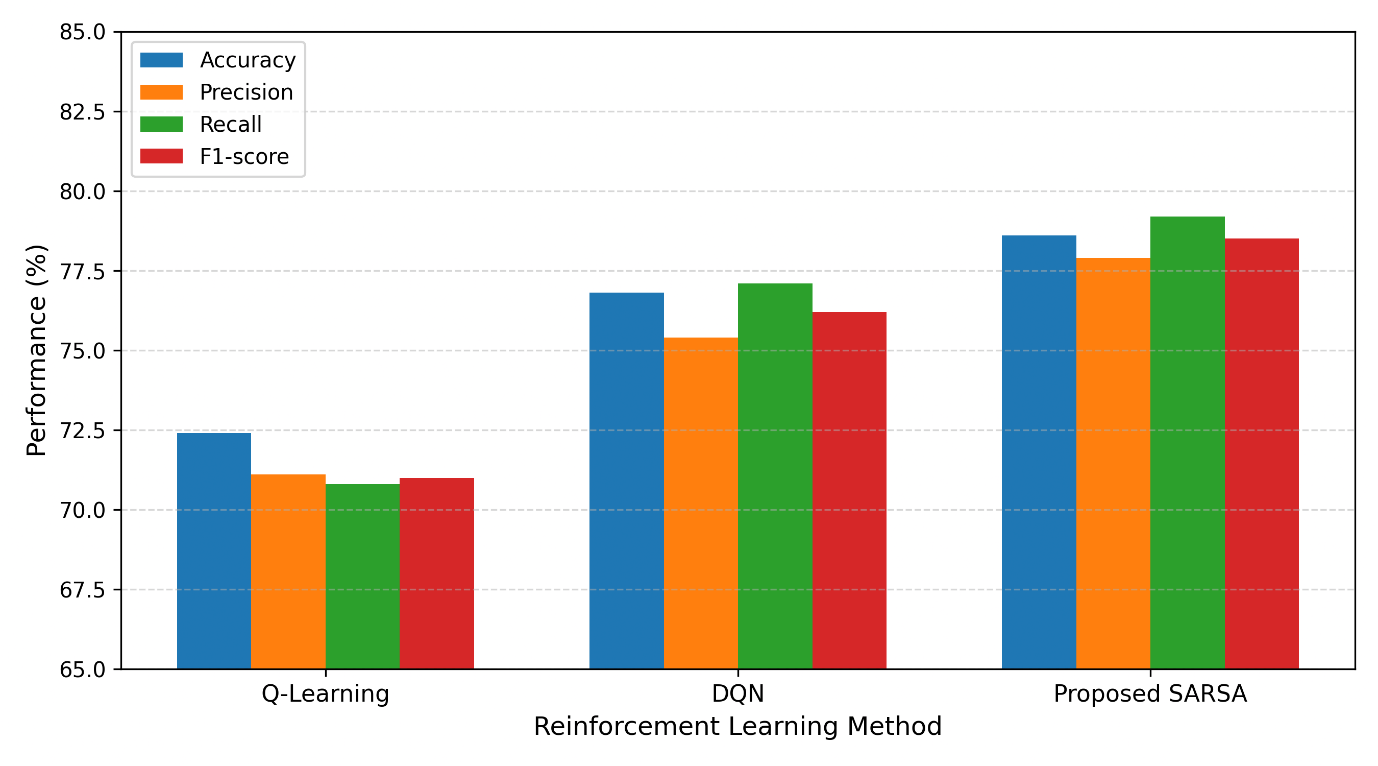


**Supplementary Figure S3. Comparative performance of reinforcement-learning-based temporal optimisation methods.**


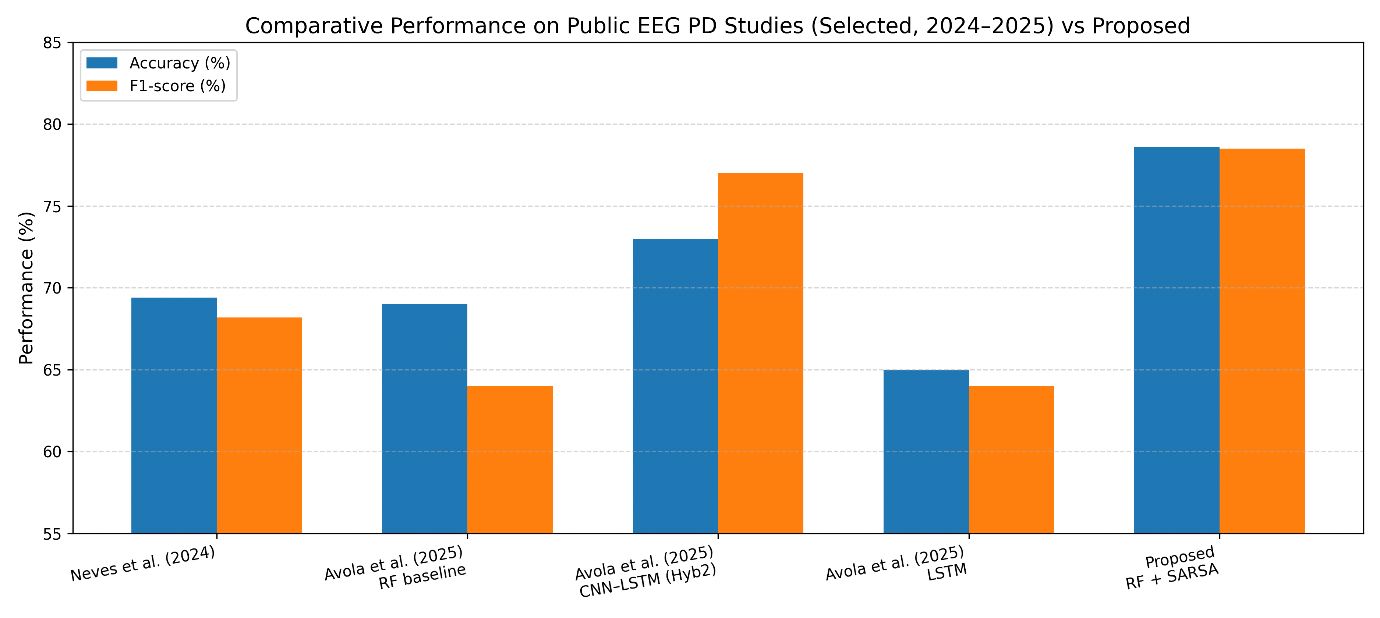
Supplementary Figure S3 compares the proposed SARSA-based refinement method with Q-learning and DQN. The SARSA-based approach gives better results across the reported evaluation metrics. The improvement is mainly seen in recall and F1-score, which suggests that SARSA provides more stable refinement of EEG window-level decisions. These results support the use of on-policy SARSA for temporal decision refinement under the subject-wise validation setting.

**Supplementary Figure S4. Comparative performance of selected public EEG-based Parkinson’s disease studies versus the proposed RF+SARSA framework.**

Supplementary Figure S4 compares the proposed RF+SARSA framework with selected recent studies on EEG-based Parkinson’s disease detection. The proposed method performs better than the listed baseline approaches in terms of accuracy and F1-score. This suggests that the model provides more reliable classification and maintains a better balance between correct detection and error control. The comparison also shows the value of SARSA-based temporal refinement, as it helps change window-level EEG predictions into more stable subject-level decisions.
